# Enhanced Performance of Next-Generation Sequencing Diagnostics Compared With Standard of Care Microbiological Diagnostics in Patients Suffering From Septic Shock

**DOI:** 10.1097/CCM.0000000000003658

**Published:** 2019-04-12

**Authors:** Silke Grumaz, Christian Grumaz, Yevhen Vainshtein, Philip Stevens, Karolina Glanz, Sebastian O. Decker, Stefan Hofer, Markus A. Weigand, Thorsten Brenner, Kai Sohn

**Affiliations:** 1 Fraunhofer IGB, Stuttgart, Germany.; 2 Noscendo GmbH, Duisburg, Germany.; 3 Department of Anesthesiology, Heidelberg University Hospital, Heidelberg, Germany.; 4 Westpfalz-Klinikum GmbH, 1, Hellmut-Hartert-Straße, Kaiserslautern, Germany.

**Keywords:** blood culture, cell-free nucleic acids, deep sequencing, molecular diagnostic techniques, sepsis, septic shock

## Abstract

**Objectives:**

Culture-based diagnostics represent the standard of care in septic patients, but are highly insensitive and in many cases unspecific. We recently demonstrated the general feasibility of next-generation sequencing-based diagnostics using free circulating nucleic acids (cell-free DNA) in plasma samples of septic patients. Within the presented investigation, higher performance of next-generation sequencing-based diagnostics was validated by comparison to matched blood cultures.

**Design:**

A secondary analysis of a prospective, observational, single-center study.

**Setting:**

Surgical ICU of a university hospital and research laboratory.

**Patients:**

Fifty patients with septic shock, 20 uninfected patients with elective surgery as control cohort.

**Interventions:**

None.

**Measurements and Main Results:**

From 256 plasma samples of 48 septic patients at up to seven consecutive time points within the 28-day observation period, cell-free DNA was isolated and analyzed by next-generation sequencing and relevance scoring. In parallel, results from culture-based diagnostics (e.g., blood culture) were obtained. Plausibility of blood culture and next-generation sequencing results as well as adequacy of antibiotic therapy was evaluated by an independent expert panel. In contrast to blood culture with a positivity rate of 33% at sepsis onset, the positivity rate for next-generation sequencing-based pathogen identification was 72%. Over the whole study period, blood culture positivity was 11%, and next-generation sequencing positivity was 71%. Ninety-six percent of positive next-generation sequencing results for acute sepsis time points were plausible and would have led to a change to a more adequate therapy in 53% of cases as assessed by the expert evaluation.

**Conclusions:**

Our results show that next-generation sequencing-based analyses of bloodstream infections provide a valuable diagnostic platform for the identification of clinically relevant pathogens with higher sensitivity and specificity than blood culture, indicating that patients might benefit from a more appropriate therapy based on next-generation sequencing-based diagnosis.

## Abstract 2

Supplemental Digital Content is available in the text.

Sepsis is a major health concern with increasing frequency and an estimated global mortality rate of 5.3 million deaths per year (1). Time is crucial in the management of septic patients and early treatment, including antibiotic administration and source control are the decisive first steps that influence patients’ outcome dramatically (2–4). Current guidelines recommend the initiation of antimicrobial therapy as early as possible and preferably within 1 hour (5). However, most of early treatments are empirical, and 46% of empirical antibiotic treatments were shown to be inappropriate and associated with 35% mortality (6). About 50% was either unnecessary or too broad spectrum, increasing the risk for resistance and toxicity. Early recognition of the infecting microorganism is therefore crucial for a targeted antimicrobial therapy, reducing side effects for the patient and improving patients’ outcome. The current standard of care blood culture (BC) however is limited by long time to positivity, low sensitivity, and low specificity.

We recently published a proof of concept study, in which the general applicability of microbial circulating cell-free DNA (cfDNA) to diagnose causative pathogens in sepsis using a significance scoring system and to identify single-gene resistances via next-generation sequencing (NGS) was demonstrated (7).

The aim of the presented study was to evaluate the performance of the NGS-based diagnostic approach with a larger cohort of patients and benchmark it by direct comparison to the current standard of care BC. Due to the limitations of BC, our findings were reviewed by an independent expert jury for plausibility, and the antimicrobial treatment regimen was reassessed for putative changes.

## METHODS

### Study Design

Data result from a secondary analysis of septic patients (*n* = 50) participating in a previously published, prospective observational clinical study of our workgroup, which was conducted in the surgical ICU of Heidelberg University Hospital, Germany between November 2013 and January 2015 (German Clinical Trials Register: DRKS00005463) (8). The focus of this primary study was the immune response to fungal infections in patients suffering from septic shock, and three patients (S16, S25, and S35) were already described with special focus on fungal infections in detail, including NGS results as heat maps within this study. All study patients or their legal designees gave written informed consent. In total 50 patients suffering from septic shock according to the criteria of the Surviving Sepsis Campaign: International Guidelines for Management of Severe Sepsis and Septic Shock 2012 were enrolled in this study (9). Treatment of patients with septic shock included early-goal directed therapy (10), elimination of the septic focus, and broad-spectrum antibiotic therapy (10–12). Blood samples were collected at sepsis onset (T0) and 1 day (T1), 2 days (T2), 7 days (T3), 14 days (T4), 21 days (T5), and 28 days (T6) thereafter. Relevant baseline data (demographic data and primary site of infection), clinical data (disease severity scores such as Simplified Acute Physiology Score II, Sequential Organ Failure Assessment [SOFA] score, Acute Physiology and Chronic Health Evaluation II score, surgical procedures, antifungal therapy, and outcome variables) as well as routine infection variables (e.g., leukocytes, *C*-reactive protein [CRP], procalcitonin, and body temperature) were collected. In addition, 20 postoperative patients following major abdominal surgery without any evidence of infection were included as controls. Routine infection variables (e.g., leukocytes, CRP, procalcitonin, and body temperature), BCs, and other clinical microbiological specimens were without pathologic findings in this group. Plasma samples from the post-surgery group were collected prior to surgery (T0), immediately after the surgical procedure (T1), and 24 hours later (T2). Two septic patients as well as three patients of the post-surgery control group were retrospectively excluded from further analyses due to technical reasons, resulting in 48 septic patients and 17 post-surgery control patients. A workflow diagram to illustrate the study design and the NGS diagnostics workflow in context with clinical data is provided in **Figure S1** (Supplemental Digital Content 1, http://links.lww.com/CCM/E364). Additional details about patients, study time points, and samples are provided in **Data S2** (Supplemental Digital Content 1, http://links.lww.com/CCM/E364). Whole blood for BC and for plasma preparation for NGS testing was drawn on the same day (different EDTA tubes). Study and control patients or their legal designees signed written informed consent. All study procedures were approved by the local ethics committee (Ethics Committee of the Medical Faculty of Heidelberg, Trial Code No. S-097/2013).

### Clinical Microbiology

At Heidelberg University Hospital BC testing is routinely performed as described previously (13). Quantification of herpes simplex virus 1 DNA and cytomegalovirus DNA from plasma or tracheal secretion was carried out via quantitative real-time polymerase chain reaction as described previously (14). Cultivation of wound swabs, catheter, and stool samples was carried out as previously described (15, 16).

### Plasma Preparation and Nucleic Acid Isolation

Plasma was prepared, and nucleic acid isolation was performed as described previously (8). If plasma volumes were below 1,000 μL after centrifugation, the respective samples were excluded (Data S2, Supplemental Digital Content 1, http://links.lww.com/CCM/E364). Contamination controls were prepared following the same procedure, and quality control steps were carried out as already described (8).

### Preparation of NGS Libraries and Sequencing

Library preparation and sequencing were carried out as previously described (7) from 1 ng cfDNA using the Nextera XT library preparation kit (Illumina, San Diego, CA) with a Biomek FXP liquid handling robot (Beckman Coulter, Brea, CA). The utilized raw data for NGS-based diagnostics are deposited in the European Nucleotide Archive under the following accession numbers: PRJEB21872 and PRJEB30958.

### Bioinformatics

Bioinformatic processing and sepsis indicating quantifier (SIQ) score calculation were carried out as already described (7). Briefly, after bioinformatic removal of human sequences and taxonomic classification of nonhuman sequences, the SIQ score provides a quantitative and probabilistic assessment of every detected microbe in the respective sample based on a noninfected control group and permits a comparison between different samples, when identically processed. After normalization of read counts to the library size, a likelihood estimate of the probability to observe a certain species in the control group is generated. Under the assumption that all read counts for a certain species are Poisson distributed based on data generated from the control group, a *p* value that assesses the likelihood for read abundances outside this Poisson distribution is calculated. This *p* value along with a species-specific factor and the normalized read abundance gives rise to the individual SIQ score of a certain species in a patient sample. Control samples from elective surgery patients, which passed the quality control restrictions were added to our database to serve as the noninfected control group. The same criteria as previously described (8) were applied to exclude species hits (**Fig. S3**, Supplemental Digital Content 1, http://links.lww.com/CCM/E364) with the following exception: thresholds for bacterial, viral, and fungal hits were 10 normalized reads.

### Evaluation Clinical Expert Panel

To assess the plausibility of the calculated SIQ scores, a panel of eight clinicians specialized in intensive care, clinical microbiology, and infectiology were asked to answer a questionnaire (**Fig. S4**, Supplemental Digital Content 1, http://links.lww.com/CCM/E364). The participants were from Heidelberg University Hospital but had no role in the study. As most positive BCs were obtained at sepsis onset, only the first three time points (T0, T1, T2) were subjected to evaluation. Every case was introduced with the patient’s anamnesis, antibiotic therapy and all results from BC and NGS at T0, T1, and T2 as well as microbiological results from other specimens, if available. The concept of the SIQ score was explained to the clinical experts as well as the visualization of the results in form of heat maps, with highest scores in dark red and low scores without coloring. Color scaling was however only comparable within the results from one patient. The experts were asked to make an overall assessment of the plausibility of reported species for the individual time point, not for each species individually and note if they deemed the overall result plausible with respect to the patient history of underlying primary diseases, surgeries, complications, and microbiological results from other specimens. Majority rules were obtained as further described in the figure legend of Figure S4 (Supplemental Digital Content 1, http://links.lww.com/CCM/E364). How NGS results and clinical data were integrated is furthermore illustrated in Figure S1 (Supplemental Digital Content 1, http://links.lww.com/CCM/E364).

## RESULTS

### Participants and Study Design

In total, 48 patients with septic shock were included in the investigation, whose characteristics are described in detail in **Table 1**. The primary septic focus was the abdomen (*n* = 43; 90%), followed by the lung (*n* = 4; 8%) as well as the genitourinary tract (*n* = 1; 2%). In 31 patients (65%), sepsis was due to postoperative peritonitis following surgery of the gastrointestinal tract (*n* = 36; 75%) or hepatobiliary surgery (*n* = 10; 21%). The overall 28-day as well as 90-day mortality was 19% (*n* = 9) and 32% (*n* = 15), respectively. The median length of ICU as well as hospital stay was 20 days and 47 days, respectively. The median SOFA score among sepsis patients was 11 at sepsis onset. Seventeen patients with elective major abdominal surgery served as uninfected controls (Table 1).

**TABLE 1.**
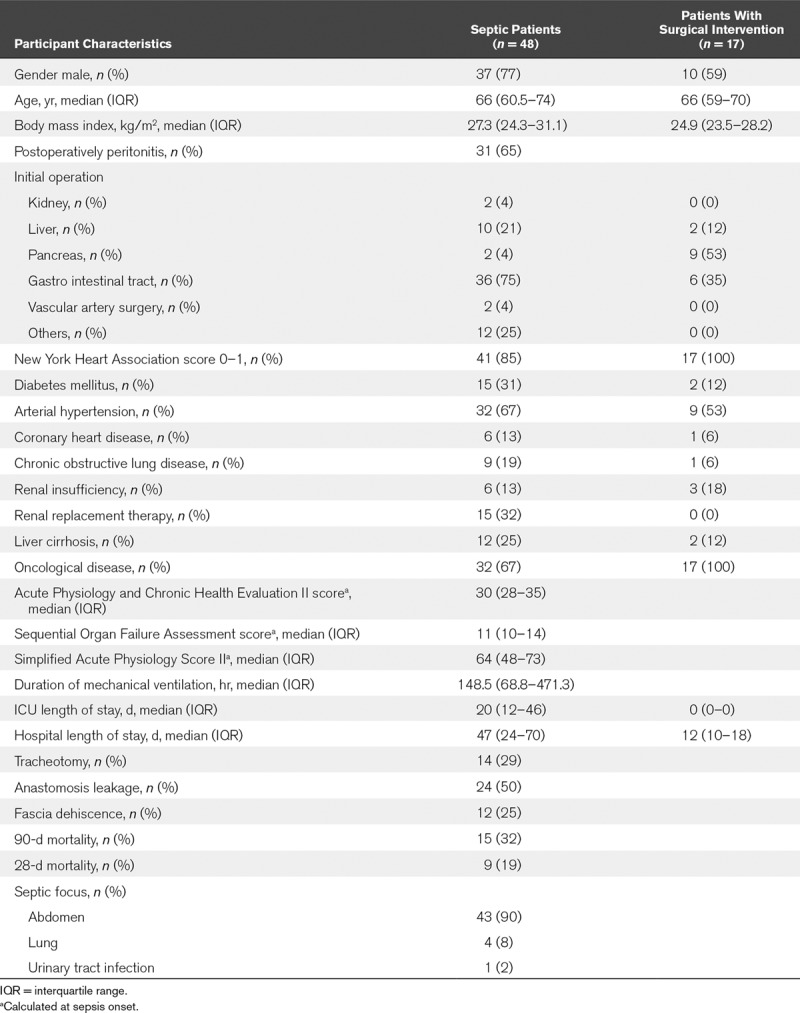
Patient and Control Group Characteristics

For the direct comparison of BC results with NGS, same day blood samples were acquired for each procedure for up to seven time points. In total, 256 blood samples were obtained from septic patients for NGS-based analysis and BC, respectively. Several samples had to be excluded as they failed to meet defined quality standards, resulting in 239 plasma samples of septic patients and 34 plasma samples of control patients for NGS. A detailed overview of patients, samples, and results is given in Supplementary Data S2 (Supplemental Digital Content 1, http://links.lww.com/CCM/E364). Results of cfDNA quantifications are shown in **Figure S5** (Supplemental Digital Content 1, http://links.lww.com/CCM/E364).

### NGS Diagnosis Yields More Positive Results Than BC

**Figure 1.**
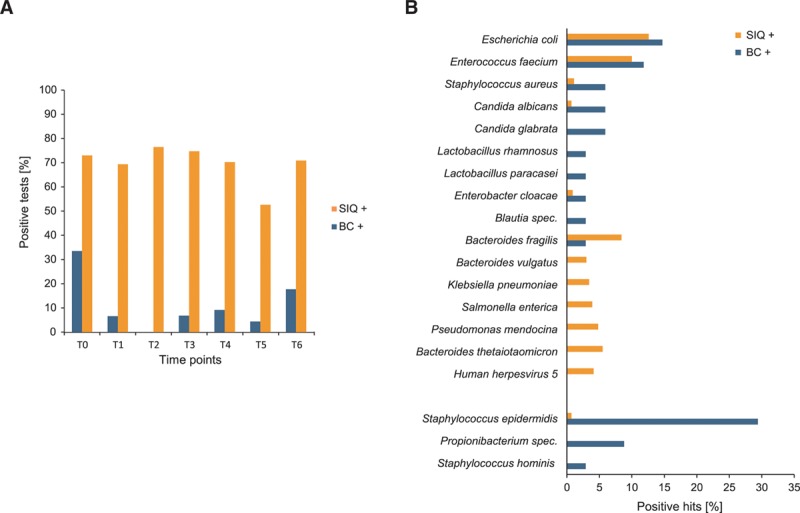
Positive results and species comparison from blood culture (BC) and sepsis indicating quantifier (SIQ) calculation. **A**, Percentage of positive results from BC (*blue*) and SIQ (*orange*) over the study time points. **B**, Percentage of species detected in the top 10 positive BC (*blue*) or SIQ (*orange*) results are displayed in decreasing order, sorted by BC results. The top two species are distributed similarly, with additional overlap in other species. A proportion of species is only observed in either one of the methods. The frequently observed potential skin contaminants Coagulase-negative Staphylococci (part of which are *Staphylococcus epidermidis* and *S. hominis*) as well as Propionibacteria are grouped at the bottom of the graph.

At sepsis onset (T0), out of 48 patients with septic shock 33% (*n* = 16) had positive BCs. NGS of 44 plasma samples at sepsis onset revealed 72% (*n* = 32) positive samples with relevant pathogens identified (**Fig. 1*A***). For the whole study period of 28 days, out of 256 BCs from 48 patients with septic shock, 11% (*n* = 29) were positive (Fig. 1*A*). In contrast, NGS of 239 septic patients’ cfDNA samples followed by SIQ score calculation yielded 169 positive results (71%) over the whole study period, which were similar for all time points (69–74%) except for T5 (52%) (Fig. 1*A*). As previously described (7, 17), approximately 98% of reads were of human origin and less than 1% of reads could be classified to microbes (**Fig. S6**, Supplemental Digital Content 1, http://links.lww.com/CCM/E364).

Out of 27 positive BCs with any NGS result, 48% (*n* = 13) identified typical skin commensals such as Coagulase-negative (CoN) Staphylococci or Propionibacteria. Three patients were found to suffer from fungemia, caused by *Candida albicans* or *Candida glabrata* (15%; *n* = 4). Predominant species in positive BCs were *Escherichia coli* (18%; *n* = 5) and *Enterococcus faecium* (15%; *n* = 4). In total, 13 unique species were identified by BC. Of these 13 species, two were fungi (*C. albicans* and *C. glabrata*) and 11 bacteria (73% gram-positive, 27% gram-negative).

By application of bioinformatics filtering and stringent decision processes (Fig. S3, Supplemental Digital Content 1, http://links.lww.com/CCM/E364), we found 169 samples with positive SIQ scores. In these 169 samples, 438 organisms and 74 unique species were identified (**Tables S1 and S2**, Supplemental Digital Content 1, http://links.lww.com/CCM/E364). Most of them (*n* = 67) were bacteria and viruses (*n* = 4), but also archae (*n* = 2) and the fungus *C. albicans* (*n* = 1). Of these bacteria, 52% were gram-negative and 48% gram-positive.

Remarkably, the two species with the highest relative abundance in BC and NGS results were similar in prevalence (**Fig. 1*B***), including *E. coli* (NGS: 12.6%; *n* = 55 and BC: 14.7%; *n* = 5) and *E. faecium* (NGS: 10.0%; *n* = 44 and BC: 11.8%; *n* = 4). Normalized read abundances of the most frequently identified species by BC and NGS show a tendency for higher abundances in patient samples in comparison to the control cohort (**Fig. S7**, Supplemental Digital Content 1, http://links.lww.com/CCM/E364).

Of 17 specimens, which were positive in BC and NGS, 7 BCs revealed CoN Staphylococci, whereas different species were identified via NGS (**Fig. S8*A***, Supplemental Digital Content 1, http://links.lww.com/CCM/E364). Of the remaining 10 positive specimens, the same species were identified by both methods in nine cases, with additional species identified either by NGS or BC in six instances, indicating a high concordance of positively identified species.

Ten specimens were solely positive by BC, of which 60% were CoN Staphylococci or Propionibacteria (Fig. S8*A*, Supplemental Digital Content 1, http://links.lww.com/CCM/E364). In four cases from positive BCs with other species than potential skin contaminants, SIQ analysis was negative. In contrast, of 169 positive SIQ results, 152 were negative by BC (**Fig. S8*B***, Supplemental Digital Content 1, http://links.lww.com/CCM/E364).

### Confirmation of NGS-Based Diagnostic Results by an Independent Expert Evaluation

The contingency table summarizes the number of positive and negative findings for BC and NGS (Fig. S8*B*, Supplemental Digital Content 1, http://links.lww.com/CCM/E364). Seventeen samples (7%) were excluded from direct comparison because these samples did not pass one of several quality filters for NGS. When taking the current gold standard BC as a reference, 17 samples were positive by BC and NGS, 10 were positive by BC only, which led to a sensitivity of 17/(17 + 10) = 62.96%. Exclusion of potential false-positive results by BC (*Staphylococcus epidermidis*, *S. hominis*, or Propionibacteria), results in 10 samples that were positive by BC and NGS and four that were positive by BC only, leading to a sensitivity of 10/(10 + 4) = 71.43%. Correspondingly, the calculated specificity was 28.30% (29.33%), positive predictive value 10.06% (5.92%), and negative predictive value 85.71% (94.29%).

**Figure 2.**
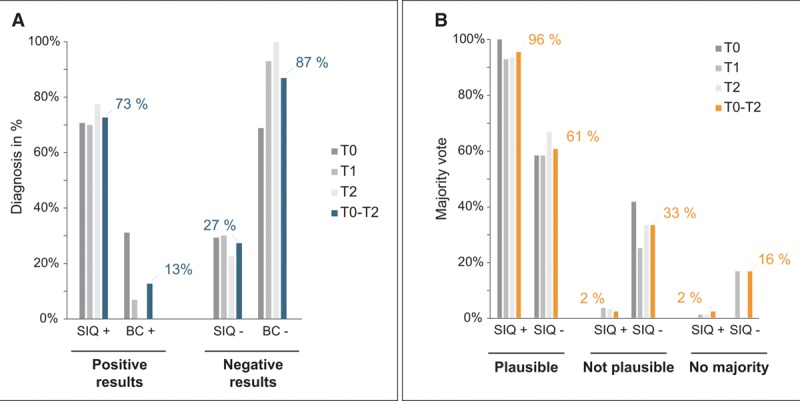
Expert evaluation of next-generation sequencing diagnosis regarding plausibility. **A**, Percentage of positive and negative results obtained for sepsis indicating quantifier (SIQ) and blood culture (BC) distributed over the first three time points (T0, T1, T2, varying shades of *gray*) or the average over T0–T2 (*blue*). **B**, Assessment of the plausibility by majority vote of the positive or negative SIQ score result over the first three time points (T0, T1, T2, varying shades of *gray*) or the average over T0–T2 (*orange*). This graph refers to question 1 in the questionnaire.

Due to the well-known limitations of BC with respect to sensitivity and specificity, BC may not represent a reliable reference to evaluate the plausibility of the calculated SIQ scores, despite being the current gold standard. We therefore held an expert adjudication assessing NGS results for the first three time points (T0, T1, T2) with respect to their clinical plausibility in the context of the patient’s medical history (Fig. S1, Supplemental Digital Content 1, http://links.lww.com/CCM/E364). For time points T0–T2 out of 121 specimens, 73% (*n* = 88) were SIQ positive and 27% (*n* = 33) were negative (**Fig. 2*A***). Of 131 corresponding BCs, 13% were positive (*n* = 17) and 87% were negative (*n* = 114) (Fig. 2*A*). Following a majority rule, SIQ results were evaluated as plausible for 96% of SIQ^+^-samples. Plausibility for SIQ^–^-samples was 61% (**Fig. 2*B***). All results of the expert evaluation are shown in **Figure S9** (Supplemental Digital Content 1, http://links.lww.com/CCM/E364), with the corresponding inter-rater agreement statistics, which were adequate for plausibility of the SIQ results (**Fig. S10**, Supplemental Digital Content 1, http://links.lww.com/CCM/E364).

### Clinical Impact of NGS-Based Diagnostics

The clinical implications of NGS-based diagnosis are exemplified for patient S3, whose detailed case description is given in the figure legend (**Fig. S11**, Supplemental Digital Content 1, http://links.lww.com/CCM/E364). NGS was able to detect the major infecting pathogen *E. faecium* for patient S3 already at sepsis onset and all later time points, in contrast to BC. The clinical experts regarded the NGS-based results as plausible and voted for a retrospective change in the antimicrobial treatment, here a de-escalation of antibiotics. Based on NGS findings, the adjustment to monotherapy would have been possible already at sepsis onset. All heat maps for all patients with integrated NGS and clinical microbiology results are shown in the **Supplemental Data File 2** (Supplemental Digital Content 2, http://links.lww.com/CCM/E365).

**Figure 3.**
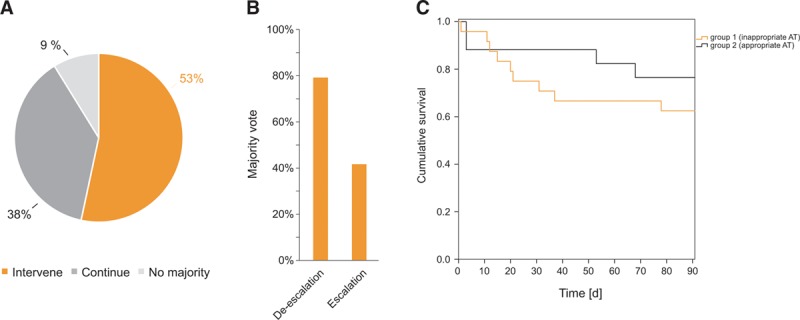
Expert evaluation of the impact of next-generation sequencing (NGS) results on the antimicrobial therapy (AT). **A**, Results from the majority vote regarding the hypothetical therapy intervention based on NGS results (question two of the questionnaire). **B**, Percentage of answers evaluated by majority vote regarding the nature of therapy intervention (question three of the questionnaire). **C**, Kaplan-Meier curve for patient’s survival concerning group 1 (inappropriate AT) versus group 2 (appropriate AT). The *orange line* indicates group 1, the *black line* indicates group 2. Patients’ survival is given as cumulative survival over a period of 90 d.

Based on SIQ score analyses the experts would have changed the antibiotic regimen in 53% of all patients analyzed in this study (**Fig. 3*A***), recommending a de-escalation of antimicrobial therapy in 80% and escalation (to include other antibiotics) in 40% (**Fig. 3*B***; and Fig. S9, Supplemental Digital Content 1, http://links.lww.com/CCM/E364). Inter-rater agreement regarding therapy was only slight to fair (Fig. S10, Supplemental Digital Content 1, http://links.lww.com/CCM/E364), which is influenced by the large size of the expert jury and potentially multifaceted views on the nature of the therapy change. Another reason is that only for a subset of patients a therapy change was advised, but all patients were included in the calculation of the inter-rater agreement. According to the expert evaluation, patients were assigned into two groups: group 1, with a retrospectively recommended antimicrobial change based on NGS results (*n* = 24) and group 2, where the majority vote recommended a continuation of the antimicrobial therapy (*n* = 17). Remarkably, 28-day as well as 90-day mortality in group 1 were higher by 13% and 14%, respectively (**Table S3**, Supplemental Digital Content 1, http://links.lww.com/CCM/E364; and **Fig. 3*C***). Furthermore, we observed a significantly increased consumption of antimicrobials during the 28-day observation period in group 1 compared with group 2 (30 vs 19 cumulated daily therapies of prescribed antimicrobials; Table S3, Supplemental Digital Content 1, http://links.lww.com/CCM/E364). Furthermore, the need for renal replacement therapies was also increased in group 1 (45.8% vs 17.6%; Table S3, Supplemental Digital Content 1, http://links.lww.com/CCM/E364).

## DISCUSSION

The overall positivity rate of BC was relatively low in this study (11%), which is in line with literature (ranging from 10% [18] up to 30% [19]). The most prevalent microorganisms detected by BC were CoN Staphylococci and *E. coli*. CoN Staphylococci represent the most common bona fide BC contaminants (20) with a false positivity rate of around 80% (21, 22). Another frequent finding in BC were Propionibacteria, also rarely representing true pathogens in sepsis (23). The low sensitivity of BC coupled with the high rate of contaminants expectedly led to poor values for specificity and positive predictive value for NGS diagnostics, whereas sensitivity and negative predictive value were reasonable.

The proportion of positive BCs was highest at sepsis onset (T0) and strongly decreased at later time points. In contrast, the rate of positive NGS results was constant over the different time points, which is in line with other DNA-based diagnostic tests (24). Our results suggest a higher sensitivity over BC and independence of antimicrobial treatment. In addition, NGS-based analyses frequently covered double-stranded DNA viruses (herpesviruses) and fungi. The accuracy, with which cfDNA reflects the dynamics of infection (7) is affirmed by the high concordance of identified species in specimens which were positive by BC and NGS and the relative distribution of the most abundant species (excluding potential contaminants) in separately positive BC and NGS samples. However, there were 10 instances, in which BC was positive while NGS was negative. Of these positive BCs, 60% revealed skin bacteria. Four of these were included by the expert evaluation, and two were assessed as plausibly negative, representing bona fide contaminants, whereas the other half was assessed as implausibly negative, together with the other four specimens positive by BC (**Table S4**, Supplemental Digital Content 1, http://links.lww.com/CCM/E364). The BC positive samples contained species such as *Enterobacter cloacae*, *E. coli*, *C. albicans*, *E. faecium*, and *S. aureus* which are usually confident indicators of bacteremia or fungemia when isolated from blood (23). In case of *E. cloacae*, *E. faecium*, and *E. coli* (S04 T0, S22 T0, S53 T0), only few reads were counted for the respective species, and no SIQ score was calculated due to the stringent filter of less than 10 normalized read counts. Although the respective species were identified, however, the threshold settings might have been too stringent, as already described for fungi (8). Another confounding factor might be the acquisition of samples, which were acquired on the same day but not always at the same time. The lack of NGS-identified reads for *S. aureus* for patient S12 T0 and T1 despite two positive BCs remains unclear. For this patient, a cecum perforation occurred as complication during surgery, which is in line with the spectrum of gut bacteria found by NGS. The expert panel’s vote regarding the NGS data for T0 was therefore plausible. The expert evaluation covered only the plausibility of NGS results, which does not imply implausibility of the BC results. Here, we can only speculate that positive *S. aureus* BCs for patient S12 T0 and T1 might be due to an *S. aureus* colonized central venous catheter (T0).

Although only 2% of positive NGS results were regarded as implausible, for negative NGS results the proportion was 33%, indicating the unsatisfactory lack of a result in context of a clinically expected infection, with any diagnostic method.

Finally, the majority of samples did not yield a positive BC result but were positive by NGS. As indicated by the comparison of top 10 species between BC and NGS separately as well as the expert panel, most of these species seem to be pathogenic organisms which are not found in BC due to its limited sensitivity. However, a number of identified species are previously described contaminants (25), which becomes especially obvious in batch effects of different kits used in the sample preparation (Fig. S7, Supplemental Digital Content 1, http://links.lww.com/CCM/E364). Although the most frequent contaminants were nonpathogenic environmental species, a certain overlap also of pathogenic species could be observed, for instance for *E. cloacae* or *Salmonella enterica* in the control cohort (Fig. S7, Supplemental Digital Content 1, http://links.lww.com/CCM/E364). As indicated in our decision tree (Fig. S3, Supplemental Digital Content 1, http://links.lww.com/CCM/E364), we aimed at not ruling out critical pathogens based on literature research, despite a certain overlap between described contaminants and pathogens. The occurrence of background signals in the control group stresses again the importance of a statistical qualifier such as the SIQ score to bioinformatically filter out contaminating sequences. Further data will lead to a continuous refinement of our databases and algorithms and render this platform even more precise.

Patient S3 illustrates the benefits of NGS-based diagnosis: 1) timely results, which were available since the earliest time point; 2) consistent results between NGS findings but also between NGS and other cultivation results; 3) stringent filtering and improved decision processes in combination with calibration to a control group led in this case to one significant species, while potential contaminants were mostly excluded; 4) the quantitative score, calculated individually for each pathogen allows for a direct comparison between different samples and is therefore excellently suited to monitor the time course of a patient; and 5) NGS results would have indicated an earlier change to a targeted monotherapy with vancomycin. As the patient recovered well, a reduction in the length of stay would have been a potential benefit of NGS diagnostics.

There were some limitations of this study. The most important limitation for a direct comparison is sample acquisition. Although this was closely matched, a split sample for NGS and BC would have been ideal which was unfortunately not possible in the clinical setup of this study. The importance of a robust and diverse control group cannot be overestimated to effectively deal with laboratory, reagent, workflow, or database contaminants. The clinical case mixture of the control cohort might however influence the test characteristics intrinsic to the usage of the SIQ score. Therefore, it is highly advisable to only compare results generated with the same control-database. Since the nature of the SIQ score is based on a Poisson distribution parameterized by the control cohort, it becomes apparent that more controls included, and a more heterogenic composition of this control cohort, will be of great benefit to the diagnostic accuracy generated by the SIQ score method since the median/mean microbial cfDNA burden in healthy people will be more accurately captured/modeled (25). In this context, our control cohort was limited regarding the number and the population type (monocentric study, only abdominal surgery). Using our workflow, results can be achieved within 24 hours upon arrival of the patient’s sample in the laboratory. However, the 24-hour turnaround time was calculated by adding the individual processing steps within a research environment and has yet to withstand real-life conditions in clinical laboratories. Further developments in real-time sequencing might reduce time-to-diagnosis to only a few hours (26). Although this time frame is currently out of range for a first-line decision on antimicrobial treatment, it is certainly compatible for a reevaluation and possible de-escalation of broad-spectrum antibiotics.

## CONCLUSIONS

Our main findings from this study are an over six-fold higher positivity rate of NGS over BC for serial samplings taken over the 28-day study period. The results of NGS diagnostics were assessed as plausible in 96% of positive SIQ results and would have led to a change in antimicrobial therapy in 53%. Despite the limited cohort size, we could observe remarkable trends in the two groups, which were retrospectively assessed in the expert evaluation as treated adequately or inadequately based on NGS results. In the adequately treated group, 28- and 90-day survival was higher and the overall use of antimicrobials was reduced, indicating the potential benefit of an adequate treatment for the patient, even without resistance identification. Therefore, we think that this method provides valuable data to support intensive care specialists in their treatment of patients with complex manifestations of sepsis.

## ACKNOWLEDGMENTS

We thank all patients, referring physicians, and study nurses who submitted samples. We would like to acknowledge Armin Kalenka, MD, Marcel Hochreiter, MD, Alexandra Heininger, MD, Cornelius Busch, MD, Götz Hofmann, MD, Christoph Eisner, MD, Sascha Klemm, MD, and Stefan Zimmermann, MD, for their valuable participation in the expert evaluation. We also acknowledge Ute Krauser for excellent technical support. Furthermore, we acknowledge Annette Sigl and Kevin Tourelle for their aid to collect and assign patient samples.

## Supplementary Material
