# Supplementary material for: Enhanced Performance of Next-Generation Sequencing Diagnostics Compared With Standard of Care Microbiological Diagnostics in Patients Suffering From Septic Shock
Source: Crit Care Med. 2019 Apr 12;47(5):e394–402. doi: 10.1097/CCM.0000000000003658 (PMC6485303; doi:10.1097/CCM.0000000000003658)
Supplement: Supplementary file 1 [file ccm-47-e394-s001.docx]

**Supplemental Data File 2: Heat map for each patient with timecourse, antibiosis and all clinical microbiology and NGS results over the study period. The heat maps are headed by a brief summary of the patient’s condition.**

S1: Pneumonia following proctocolectomy due to colon cancer.

**
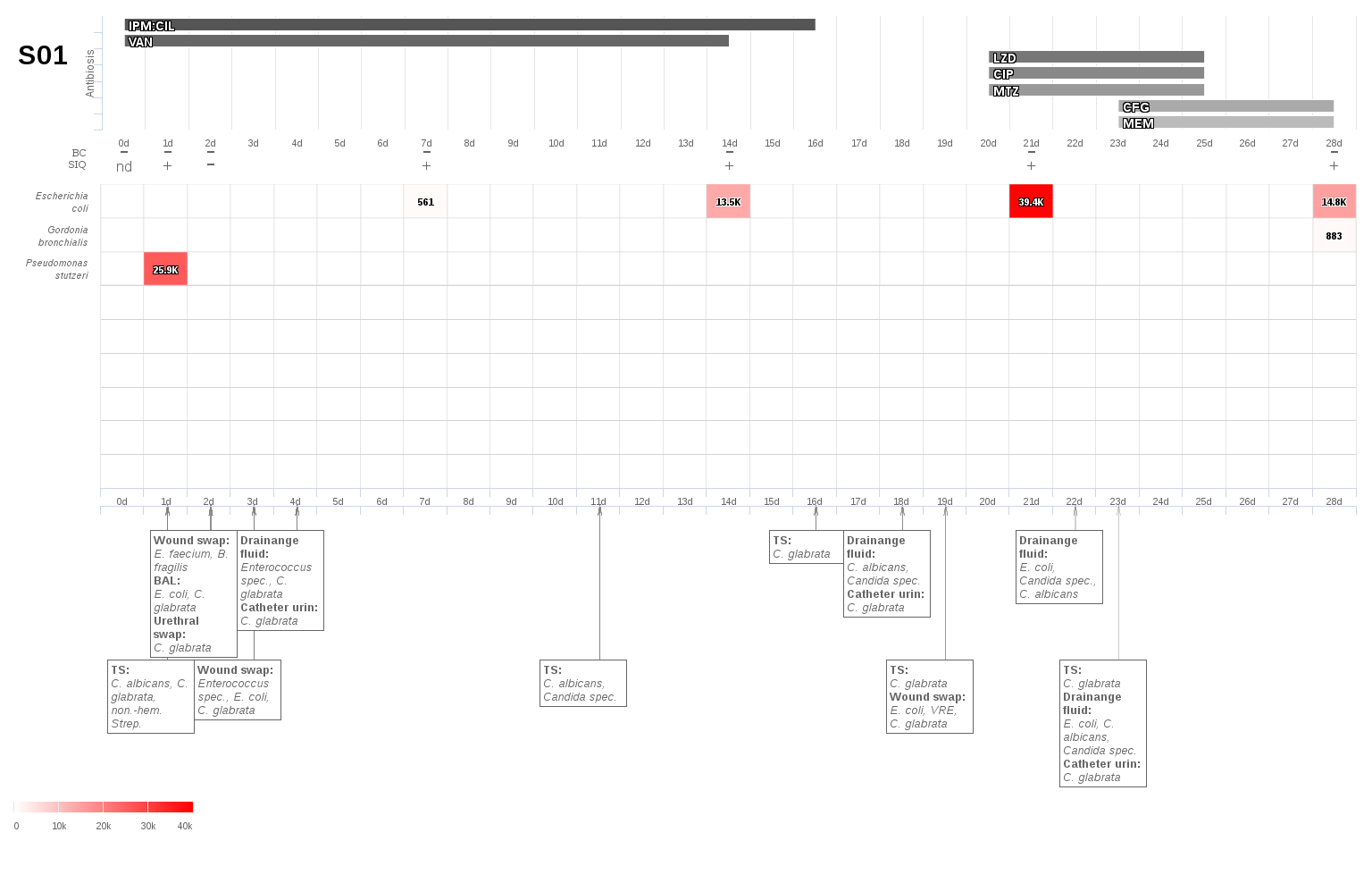
**

S2: Multiple intraabdominal abscesses due to colon necrosis following untwisting of a volvulus.

**
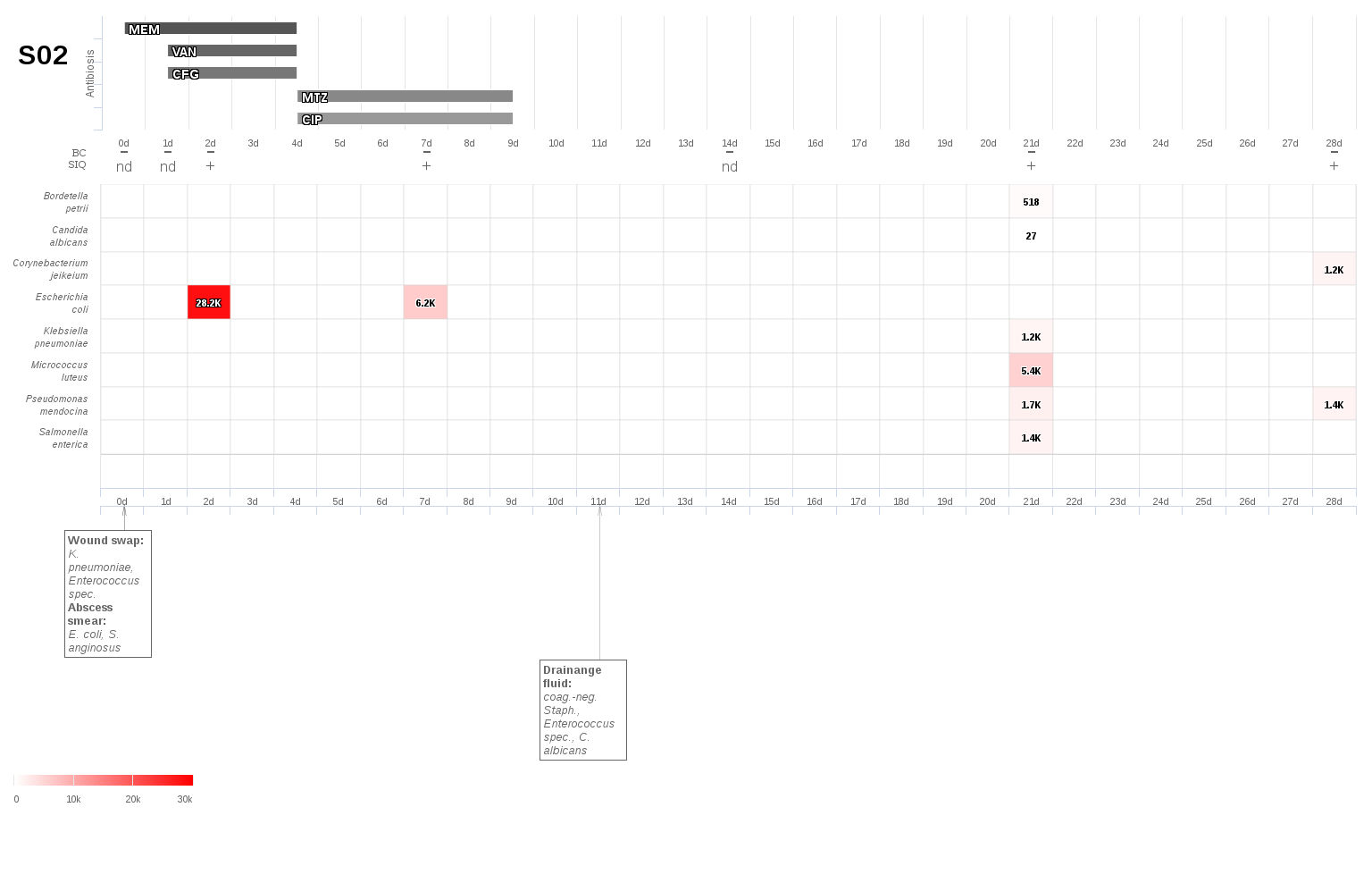
**

S3: Anastomotic insufficiency and infected hematoma following gastrectomy.

**
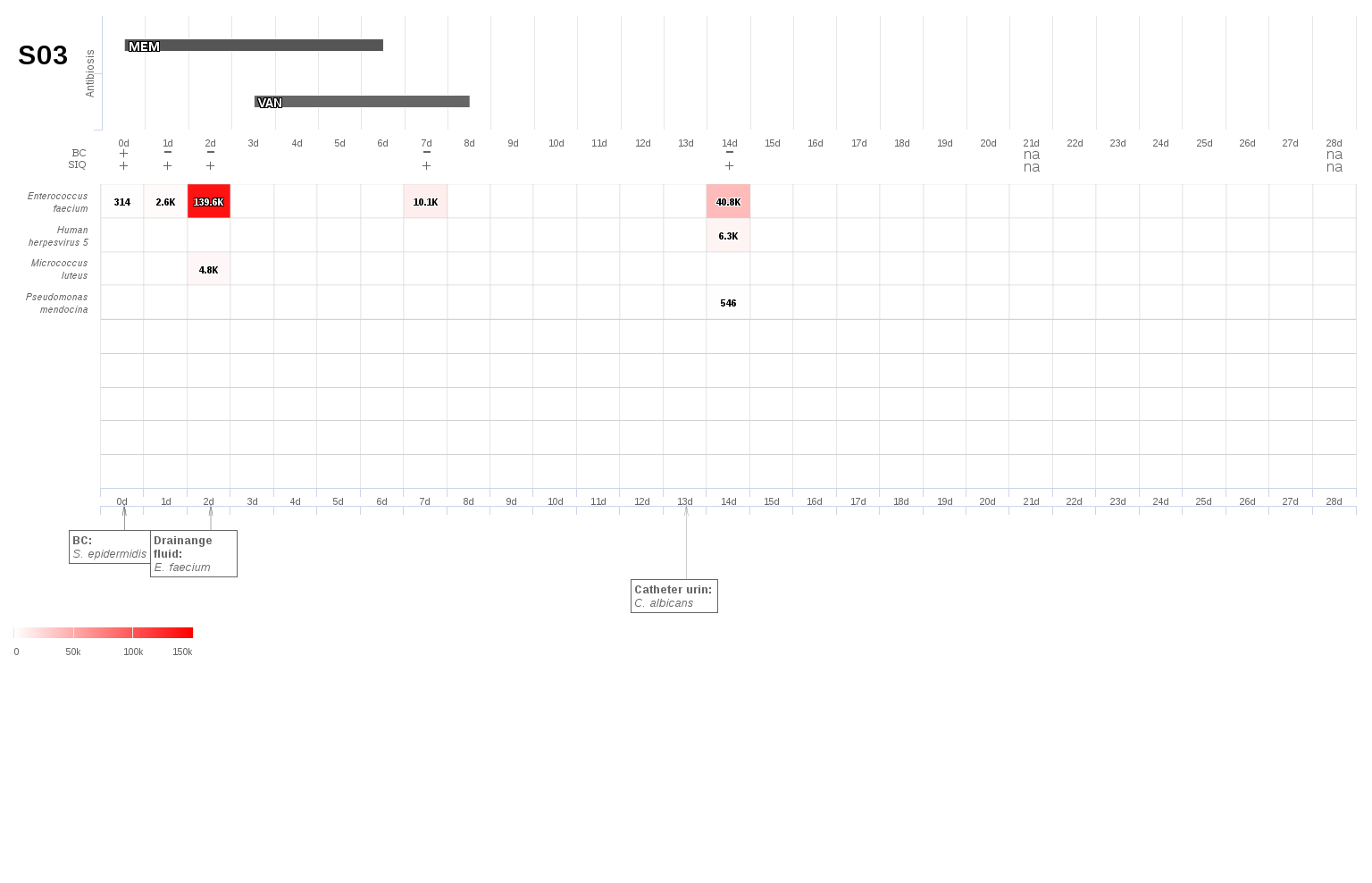
**

S4: Pneumonia following adhesiolysis of an obstructive ileus.

**
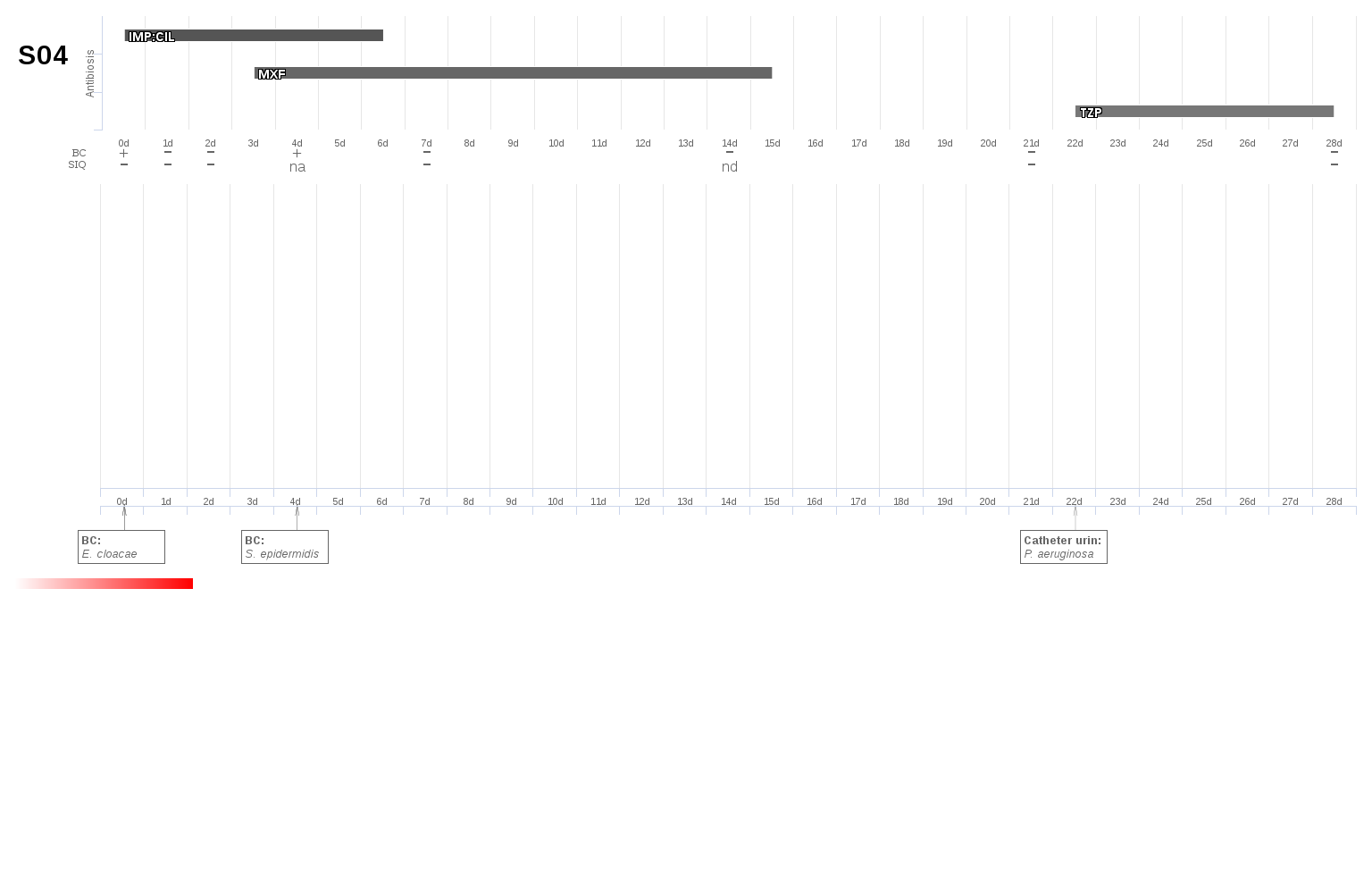
**

S5: Diffuse peritonitis with multiple abscesses following perforation of a sigmoid diverticulitis.

**
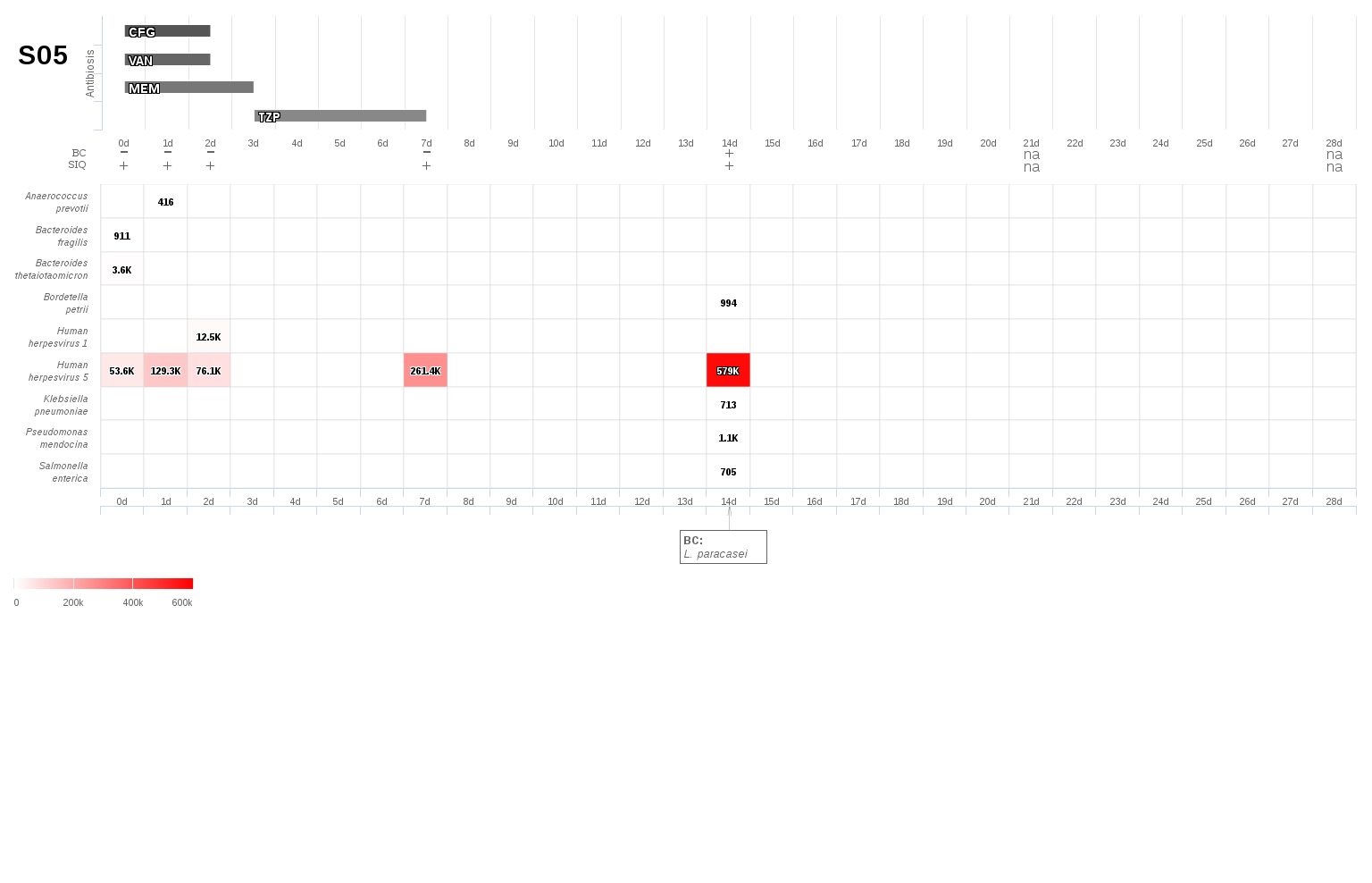
**

S6: Anastomotic insufficiency of the pancreaticojejunostomia with diffuse peritonitis following partial pancreaticoduodenectomy due to a cholangiocarcinoma.

**
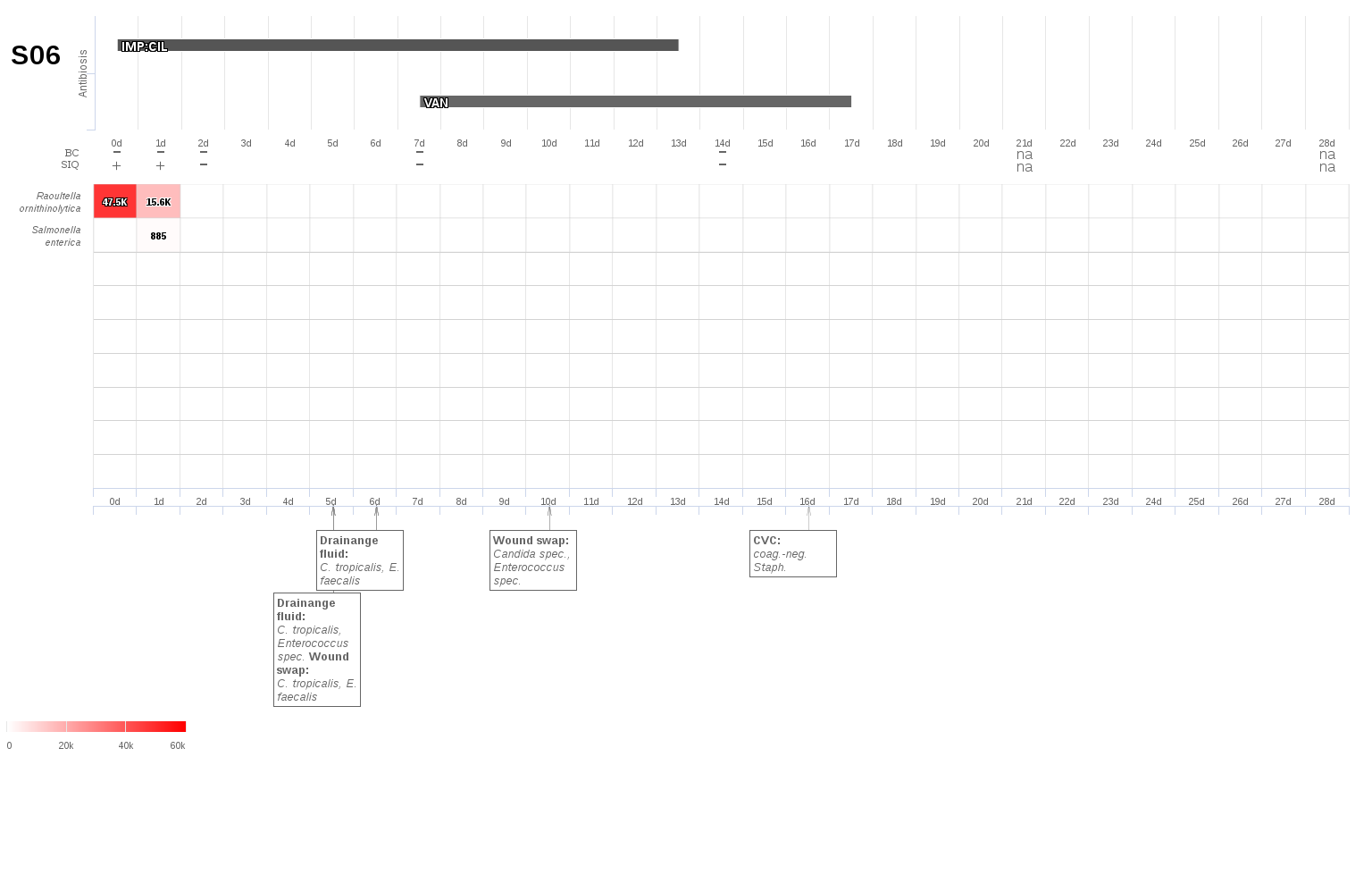
**

S7: Insufficiency of the esophagojejunostomia following total gastrectomy due to gastric cancer.

**
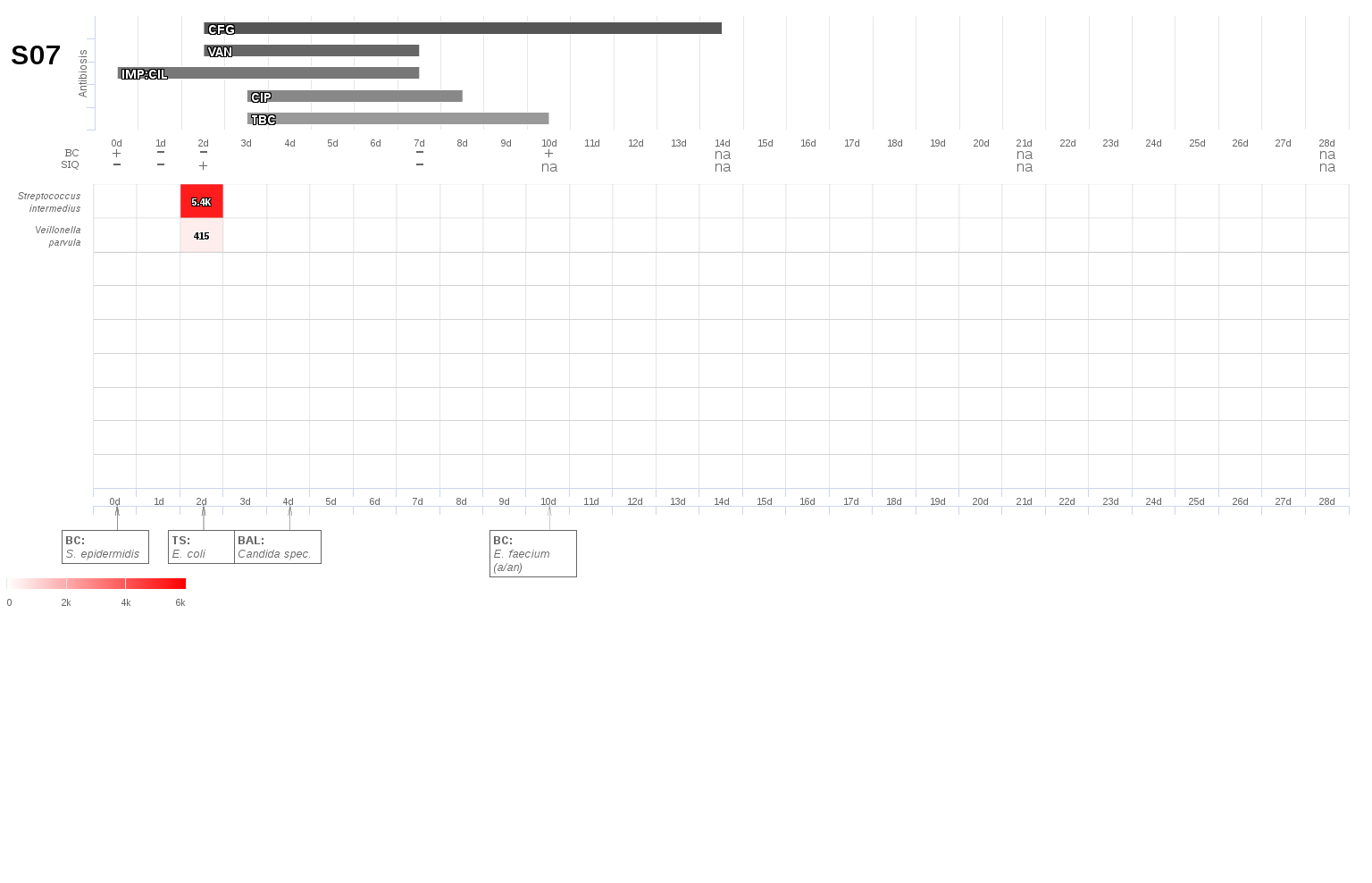
**

S8: Anastomotic insufficiency following esophagectomy due to esophageal cancer.

**
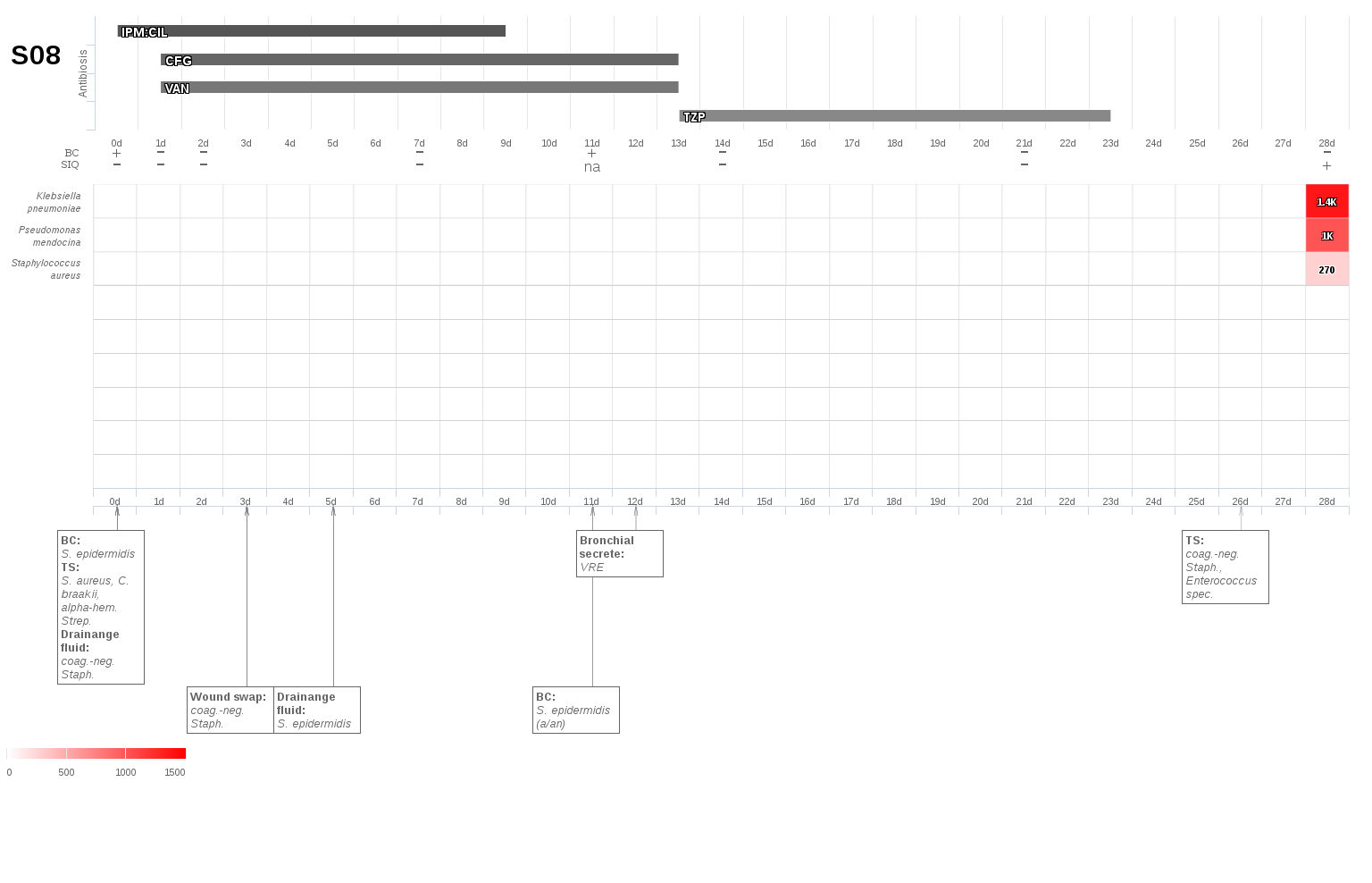
**

S9: Infected bilioma due to a biliary leakage following liver resection.**
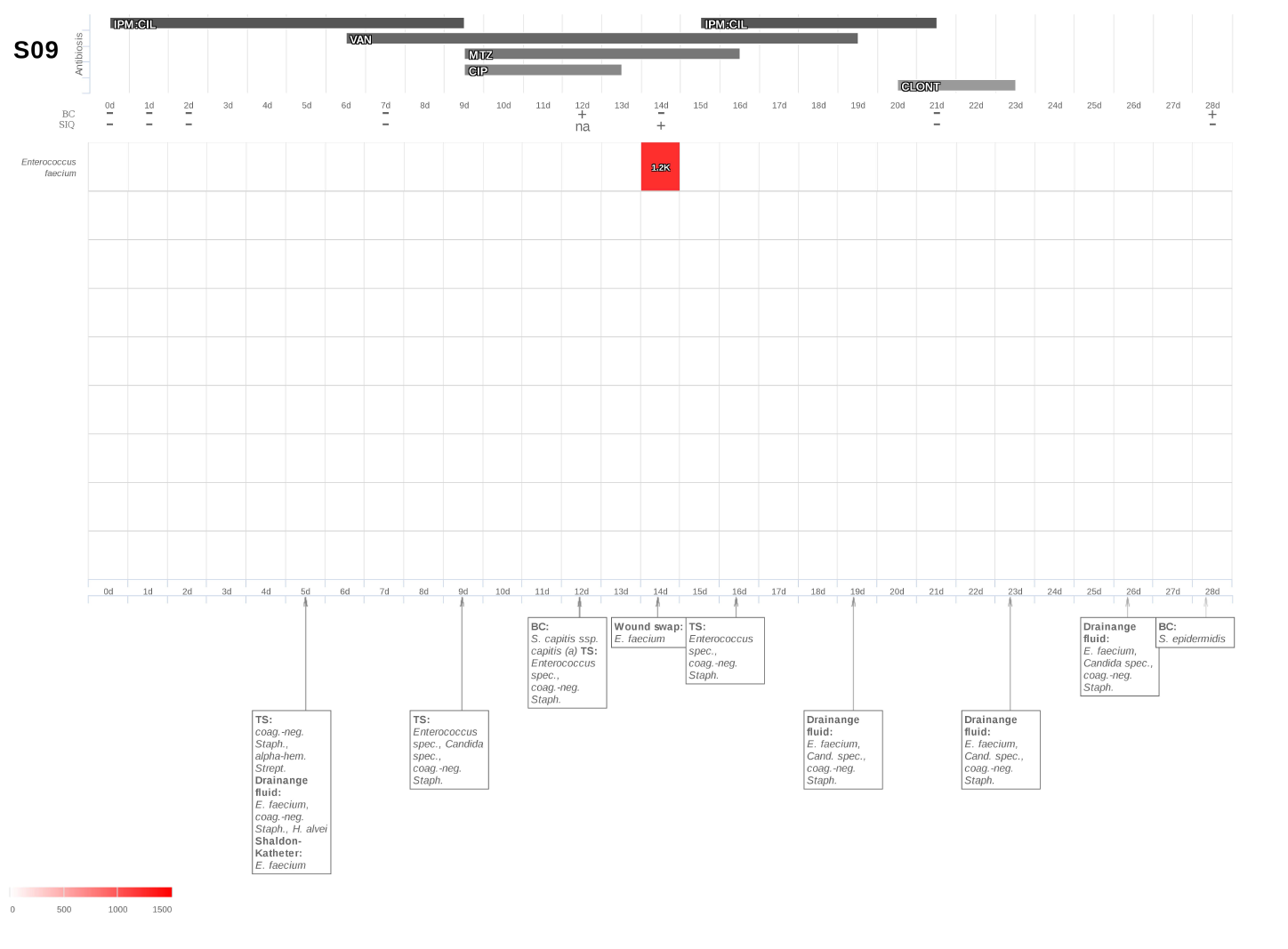
**

S10: Leakage of an ileostomy with accompanying diffuse peritonitis following bowel resection due to a liposarcoma

**
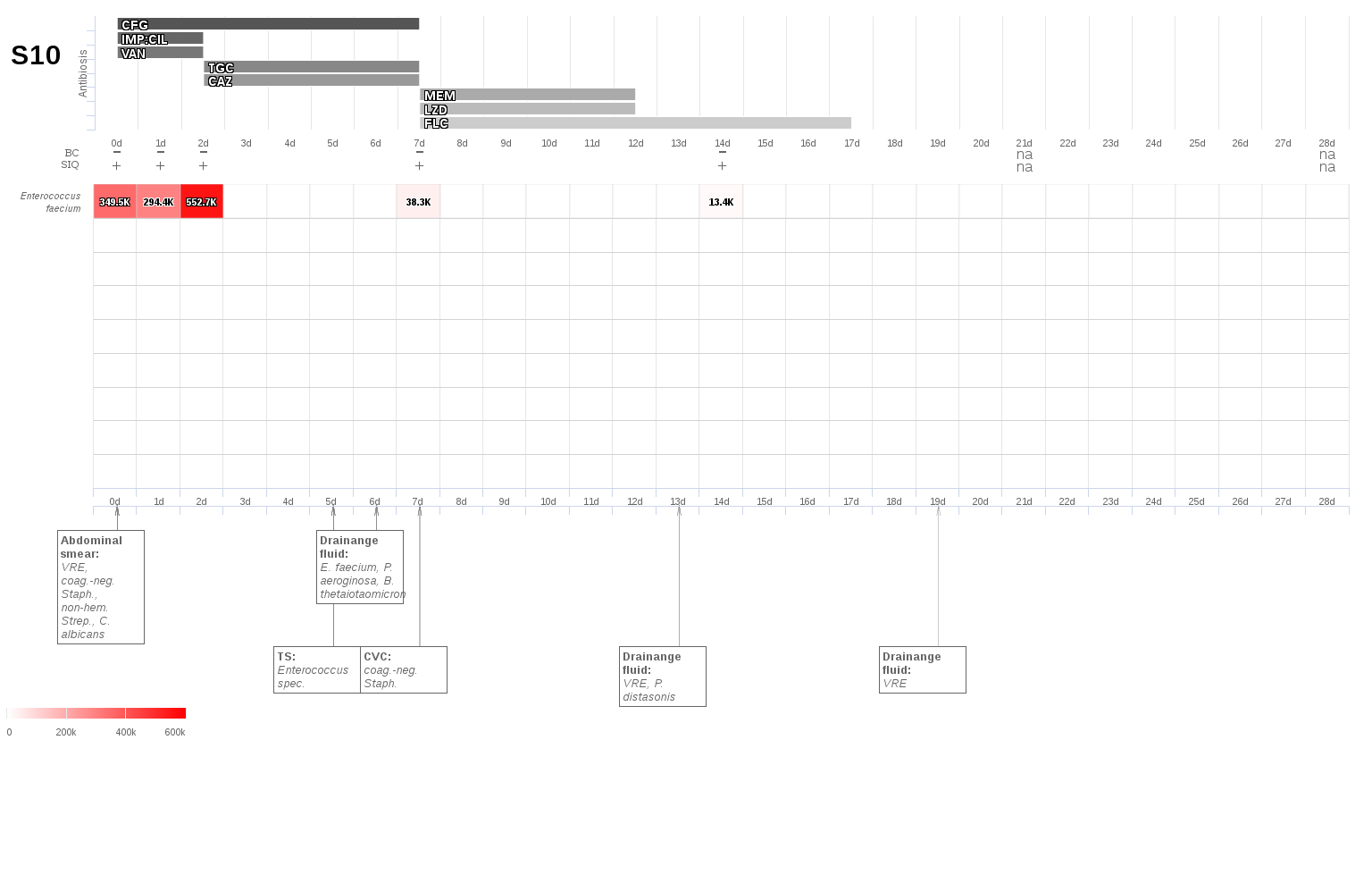
**

S11: Anastomotic insufficiency following pylorus-preserving pancreatoduodenectomy due to pancreatic cancer.

**
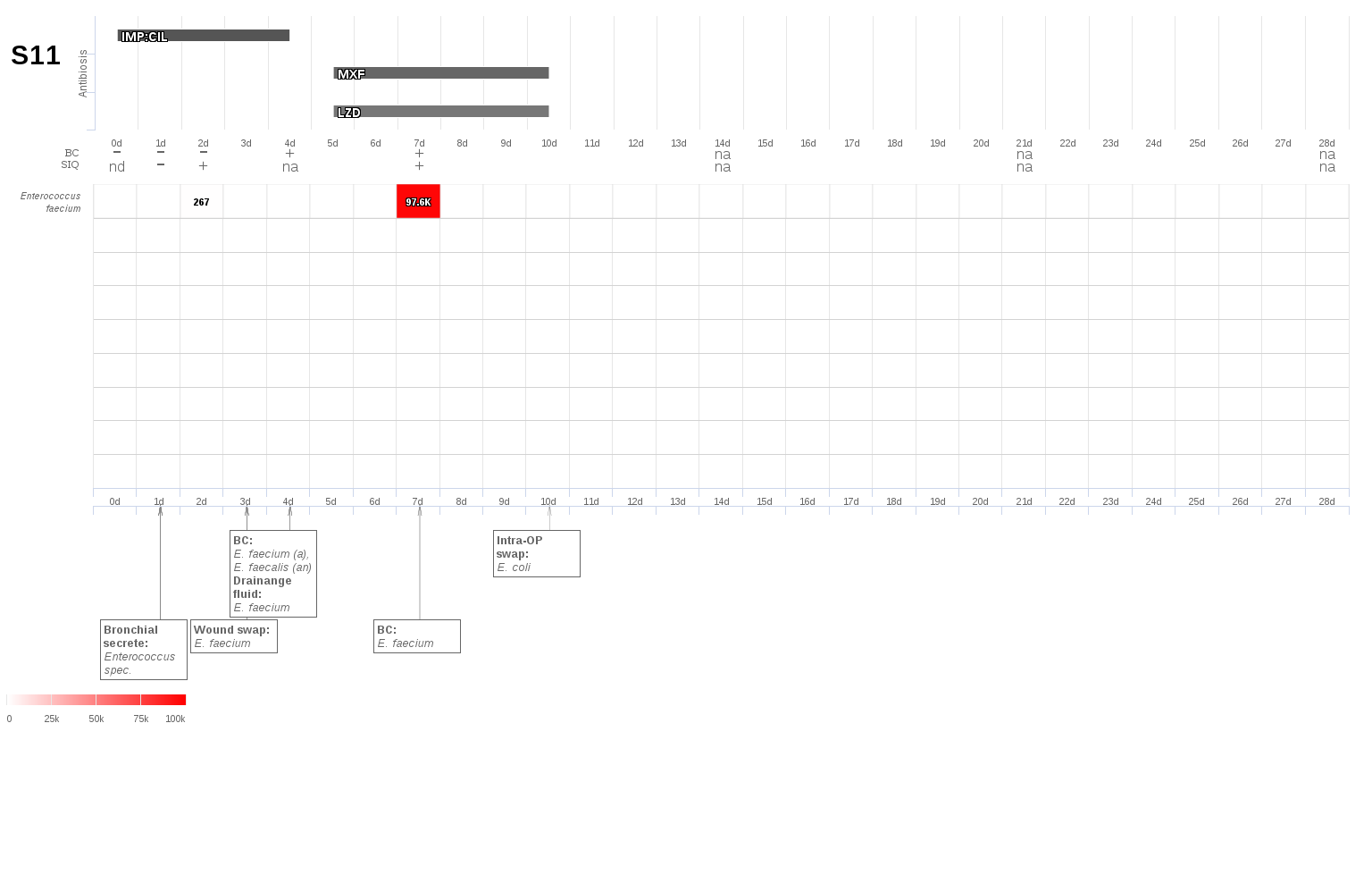
**

S12: Cecal perforation following liver resection due to a hepatocellular cancer

**
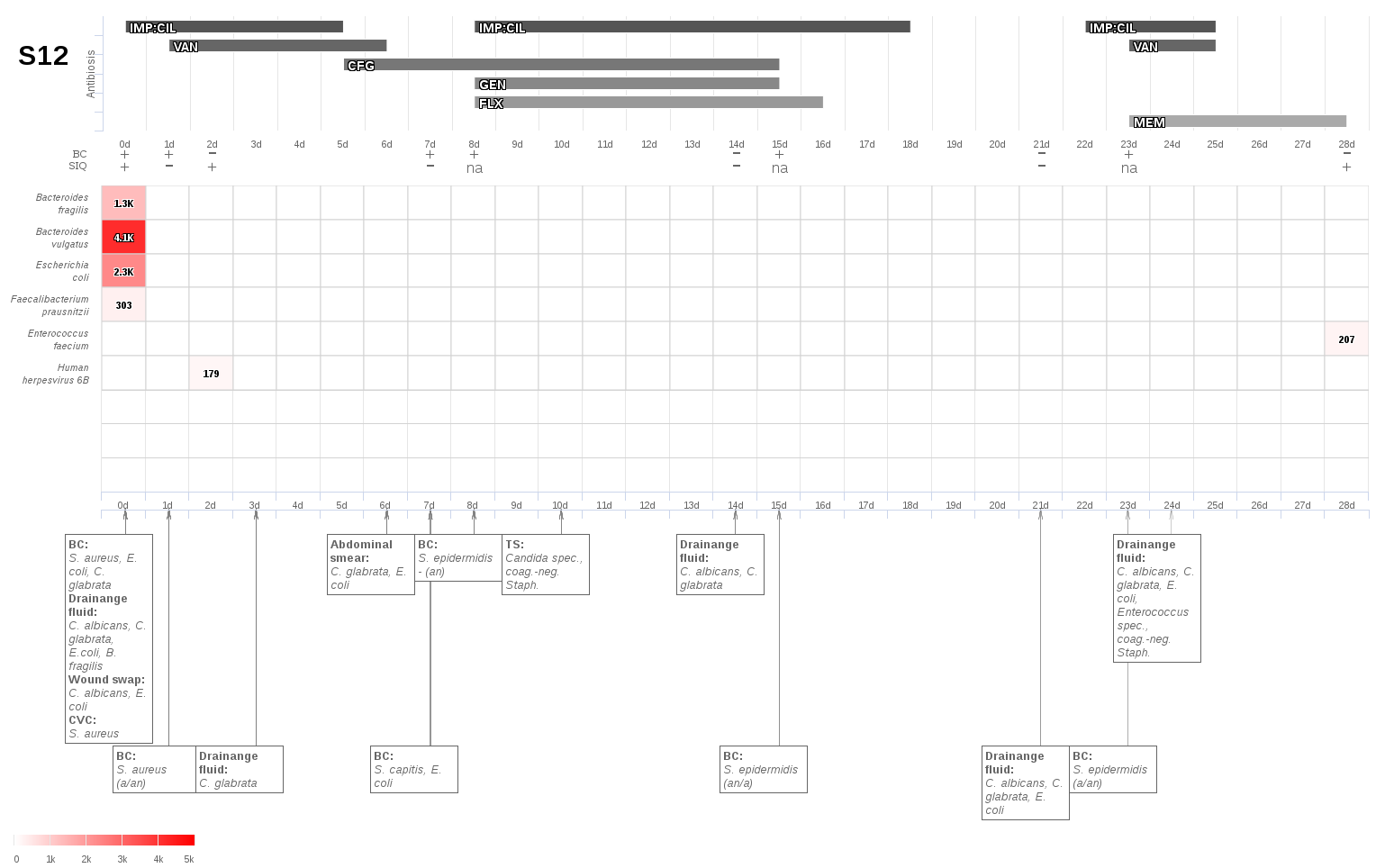
**

S13: Anastomotic insufficiency following sigma resection.

**
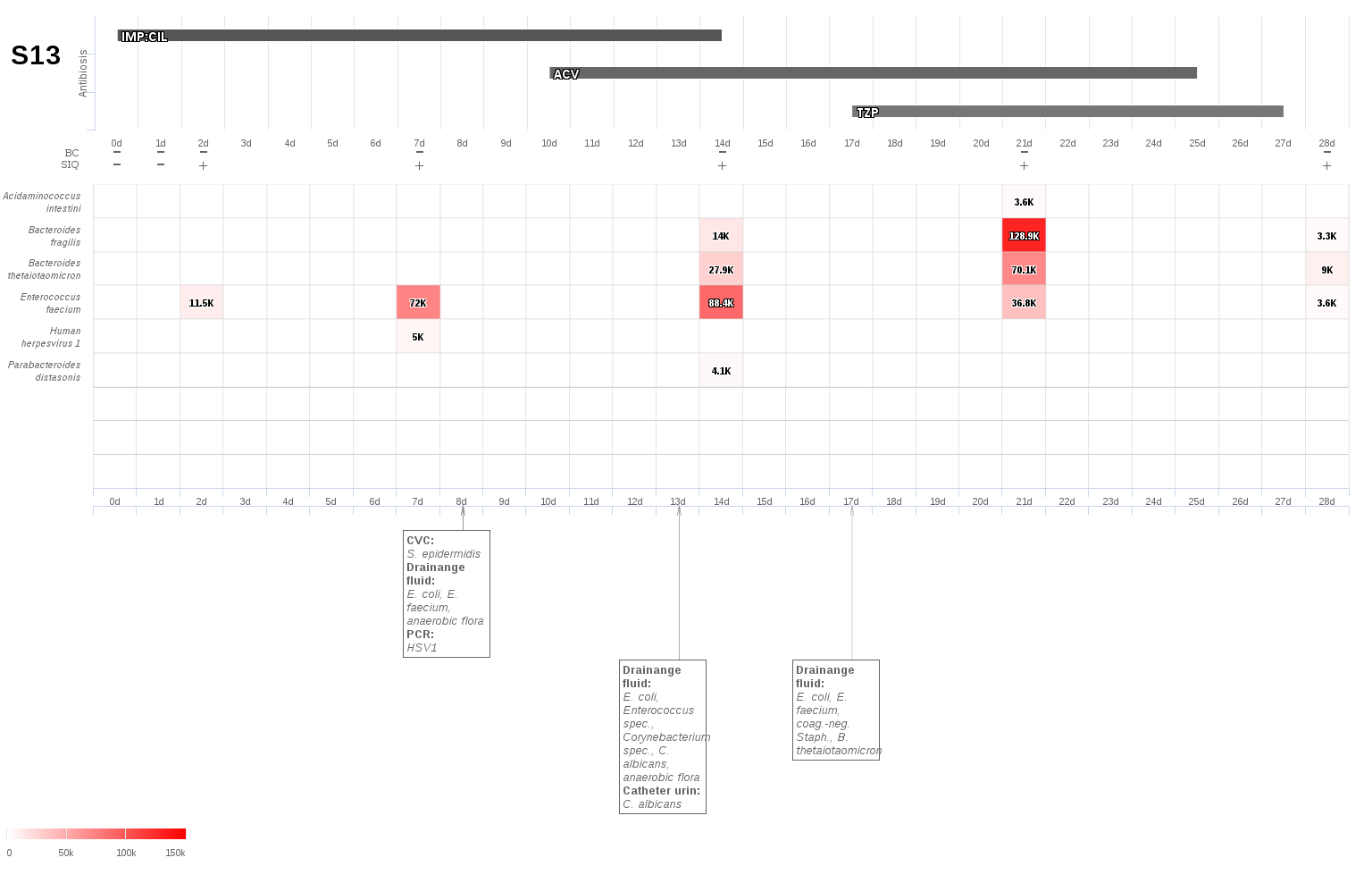
**

S14: Small bowel perforation following liver resection.

**
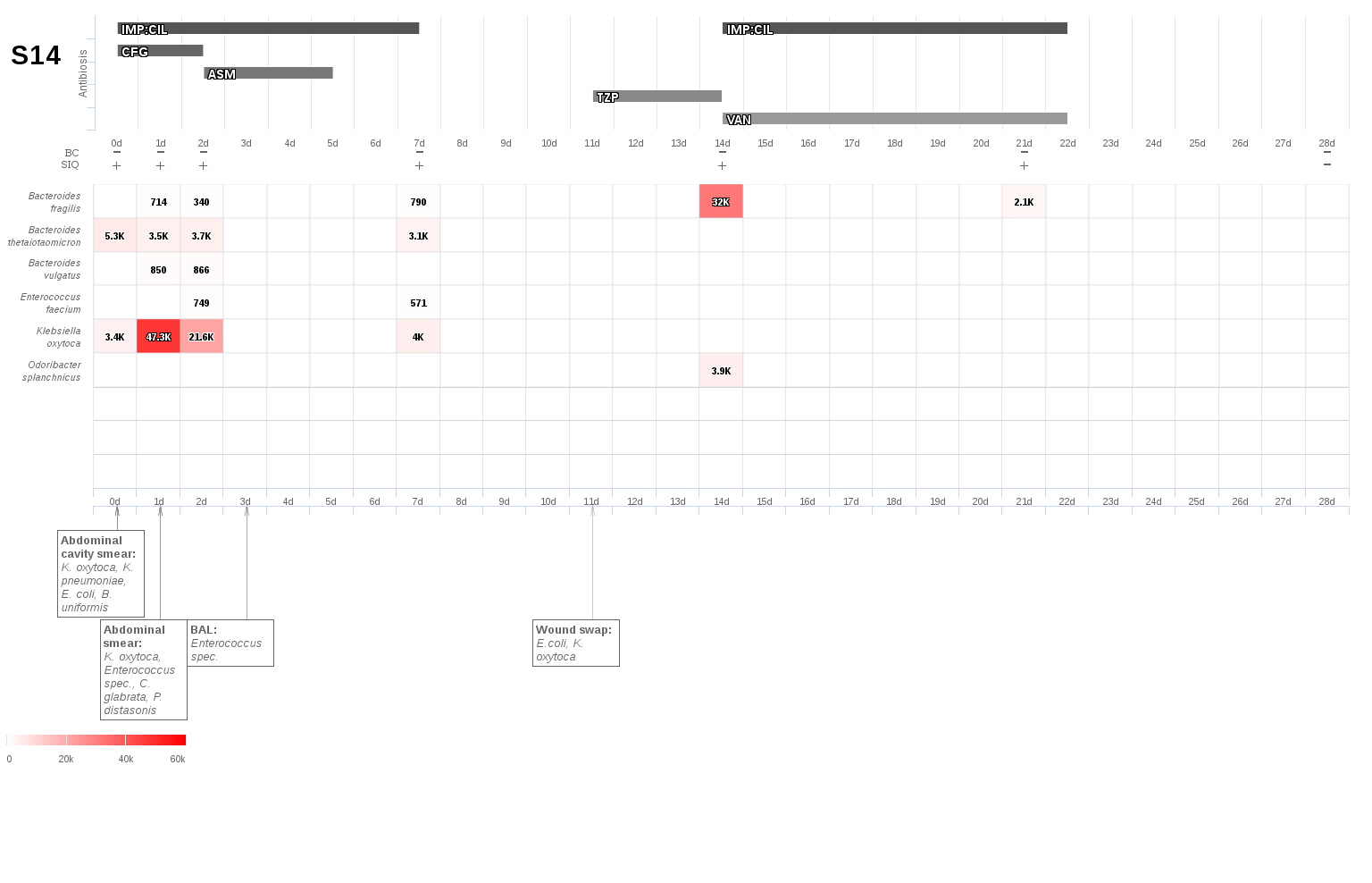
**

S15: Small bowel ischemia due to a volvulus.

**
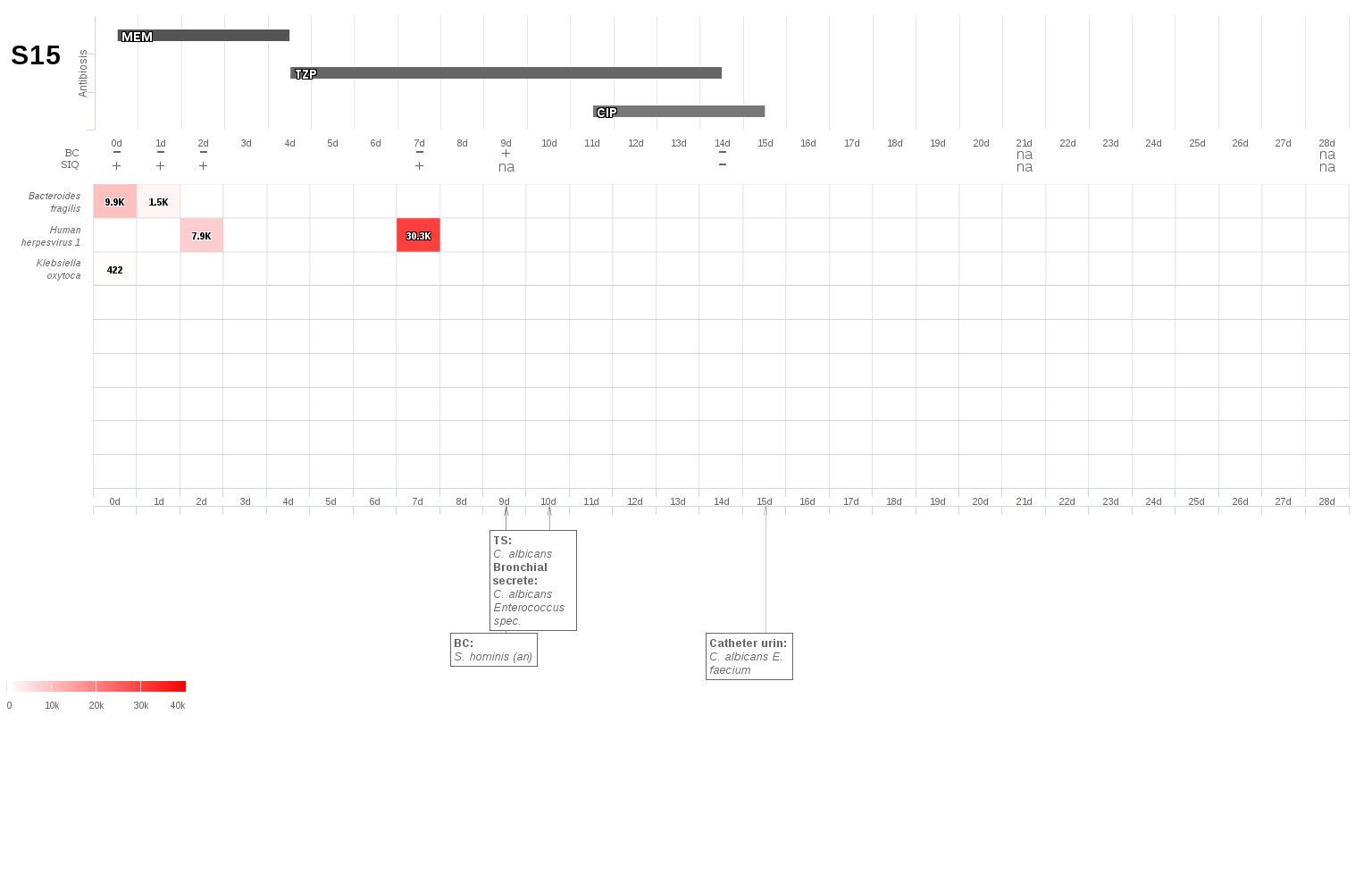
**

S16: Duodenal ulcer perforation following choledochotomy and total gastrectomy due to cholangiocarcinoma.

**
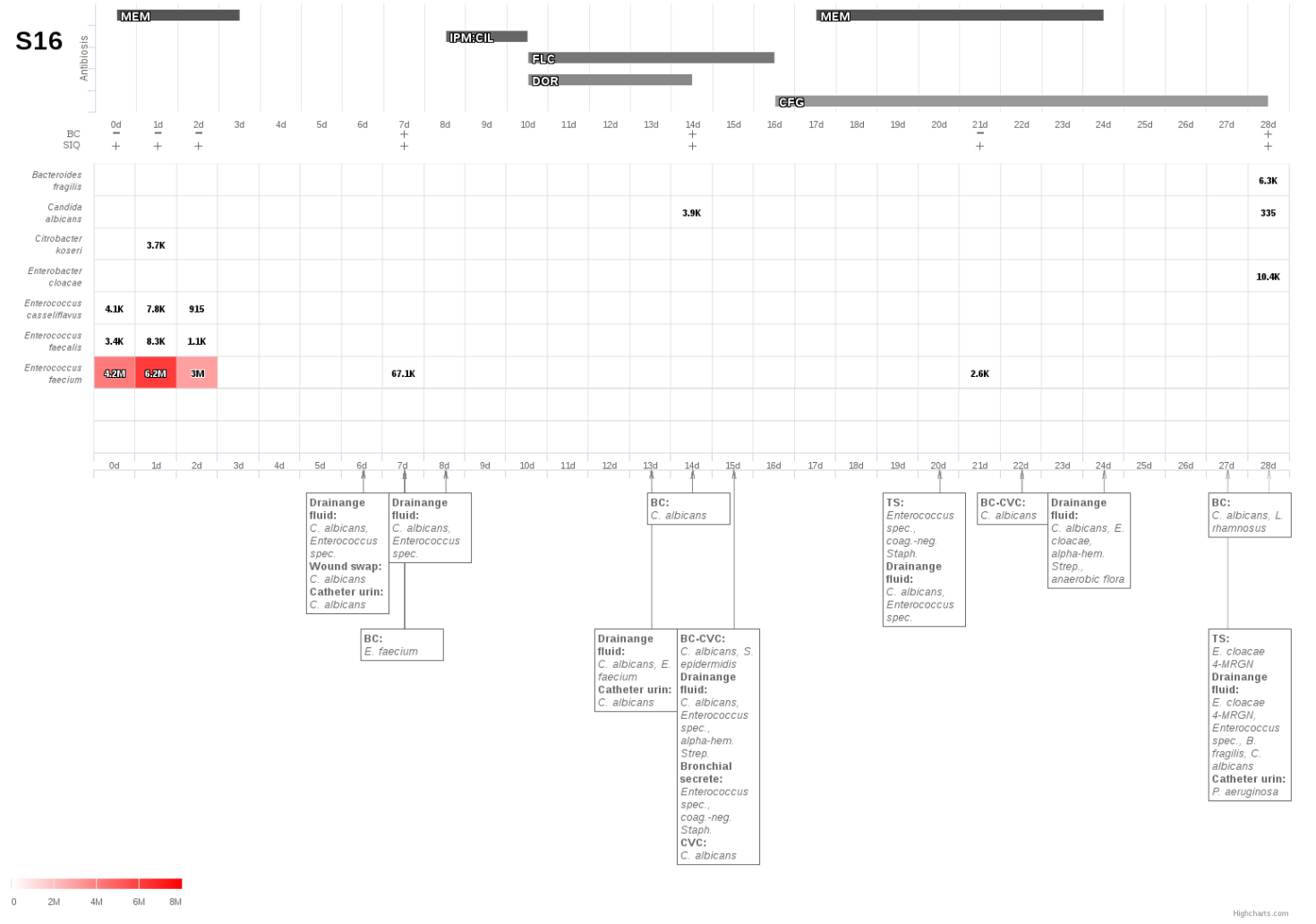
**

S17: Sigmoid perforation due to sigmoid diverticulitis.

**
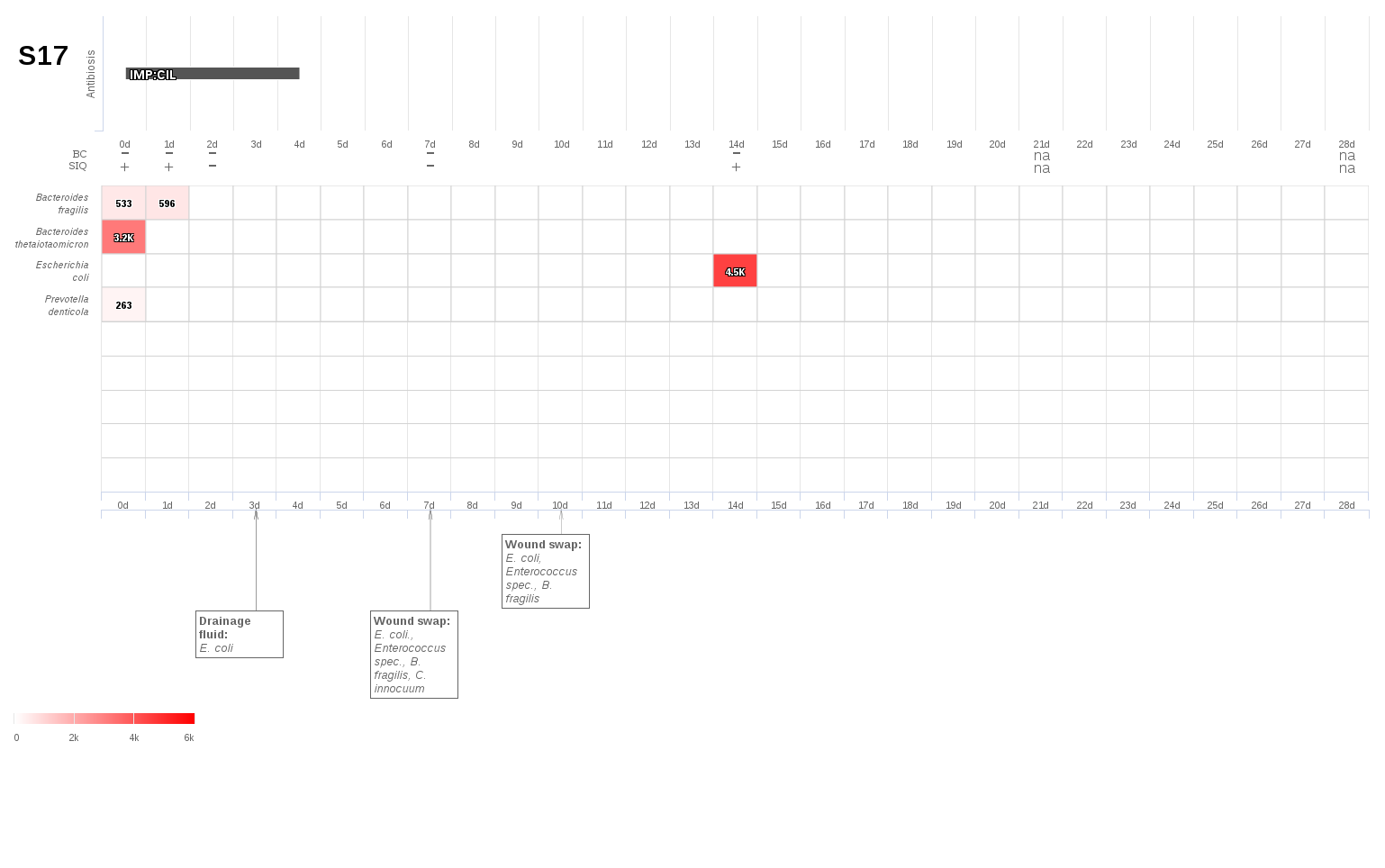
**

S19: Toxic megacolon with perforation due to a pseudomembranous colitis.

**
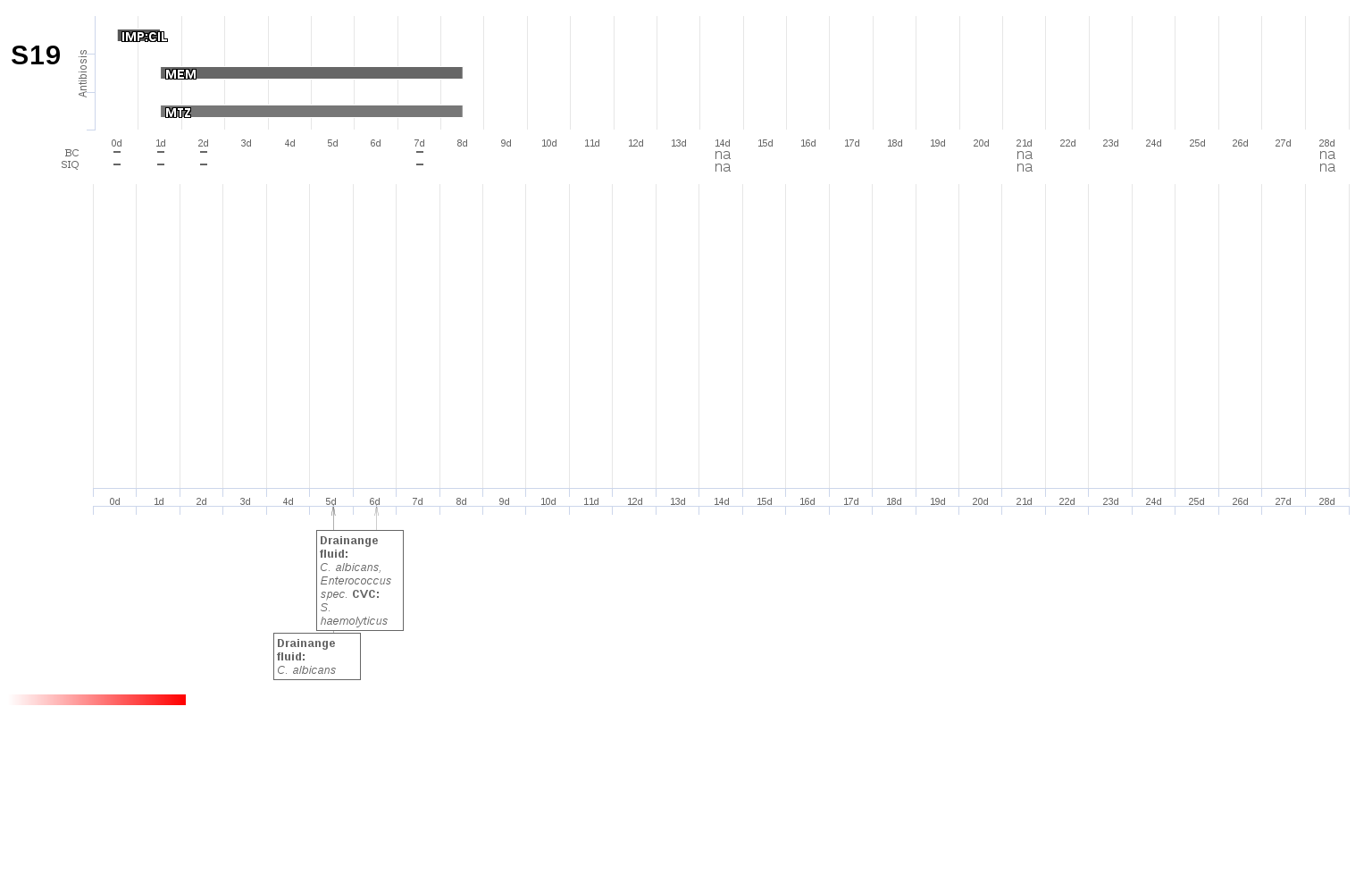
**

S20: Urosepsis due to ureteral calculus.

**
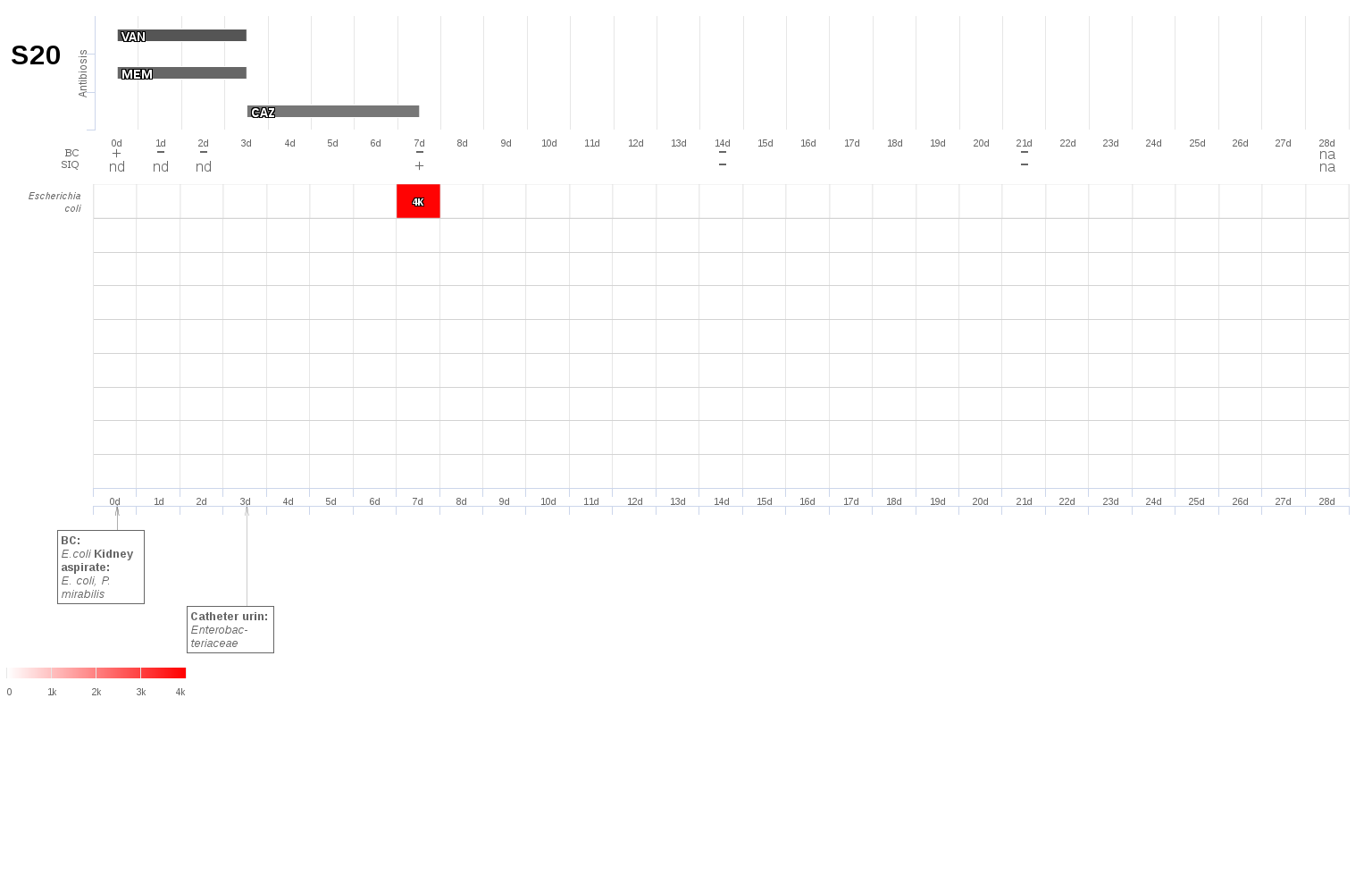
**

S21: Jejunal perforation due to a mechanical ileus.

**
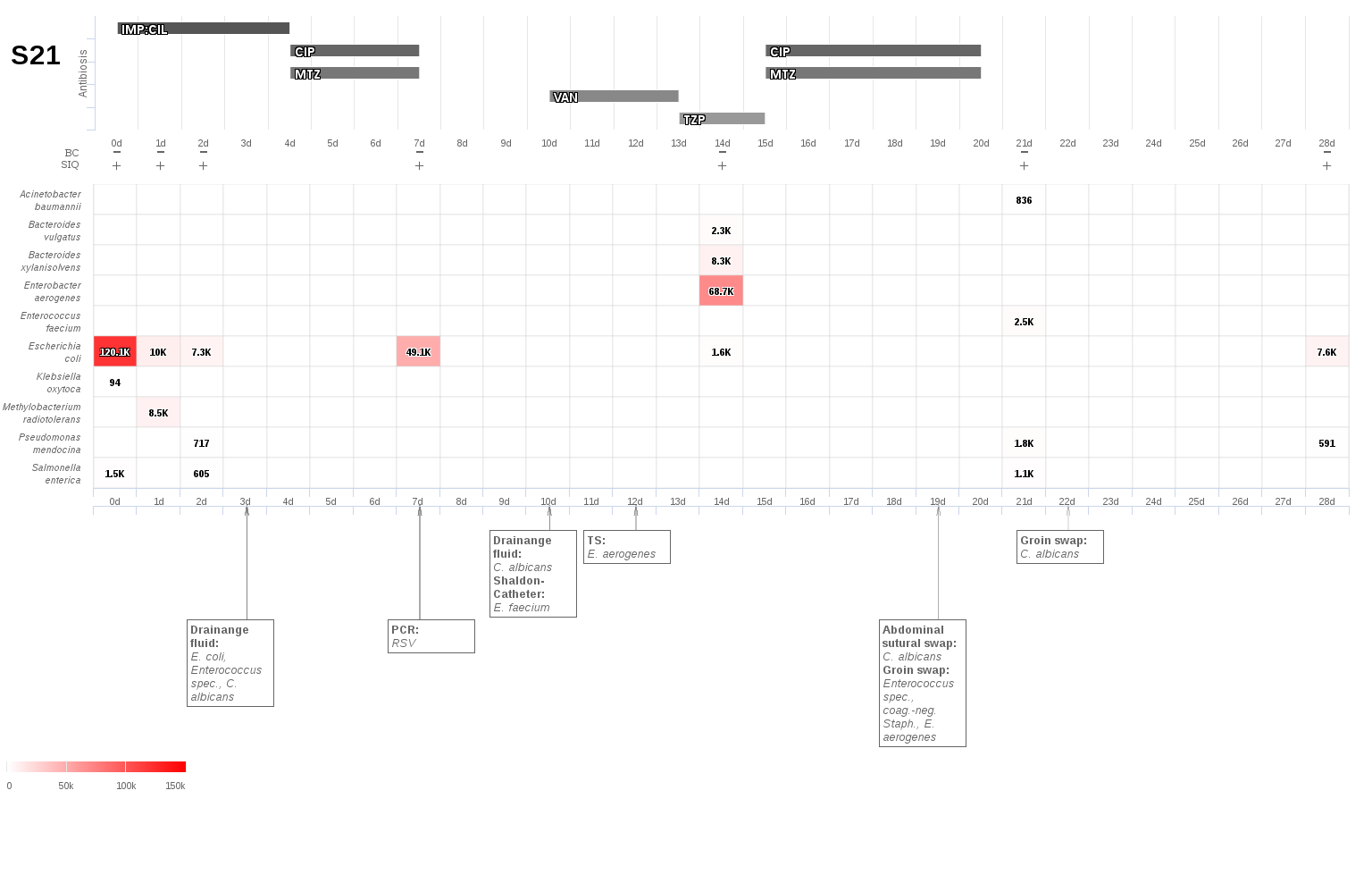
**

S22: Anastomotic insufficiency with diffuse peritonitis following hemicolectomy with ileotransversostomy

**
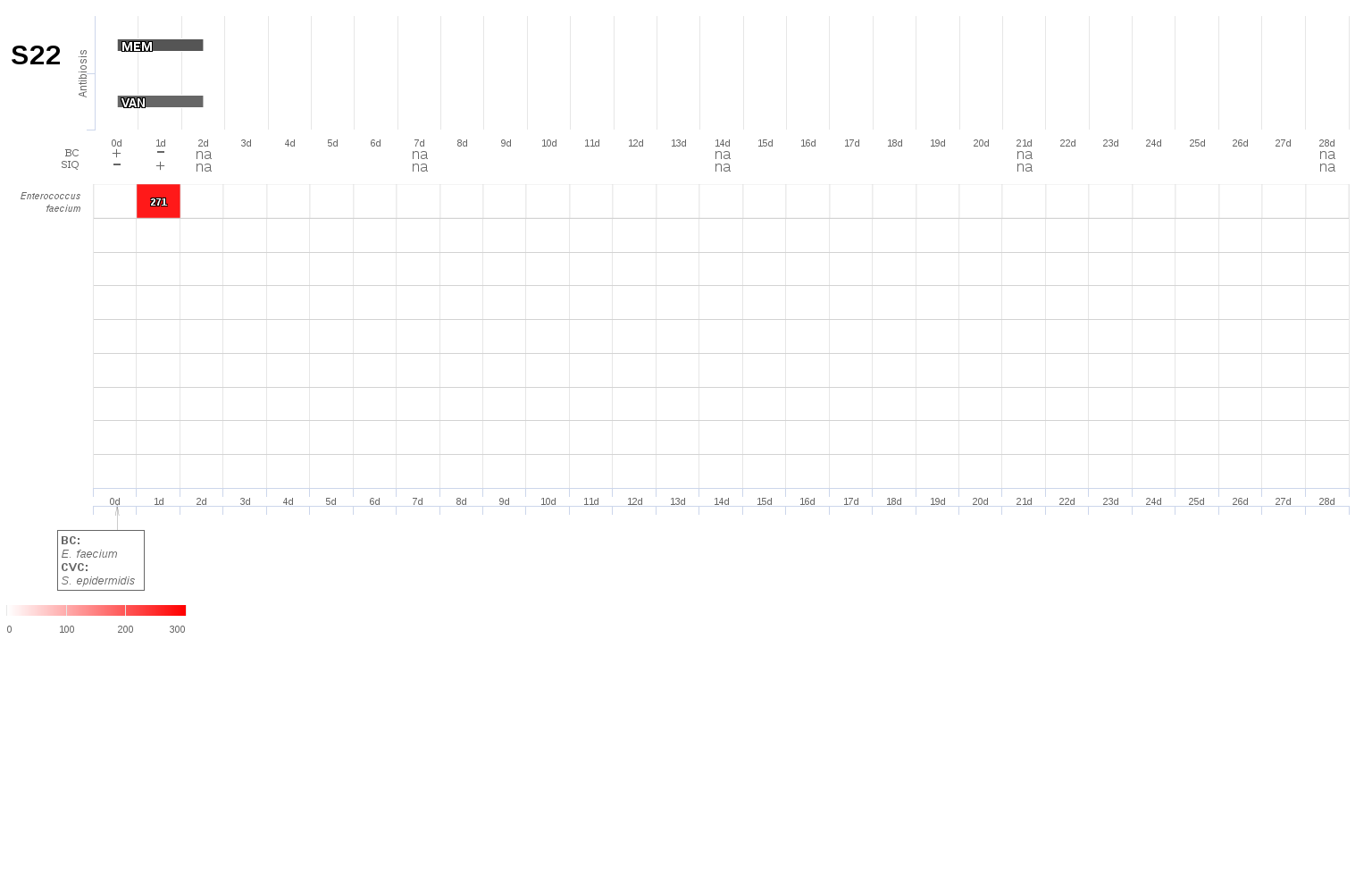
**

S23: Cecal perforation with diffuse peritonitis and acute cholecystitis.

**
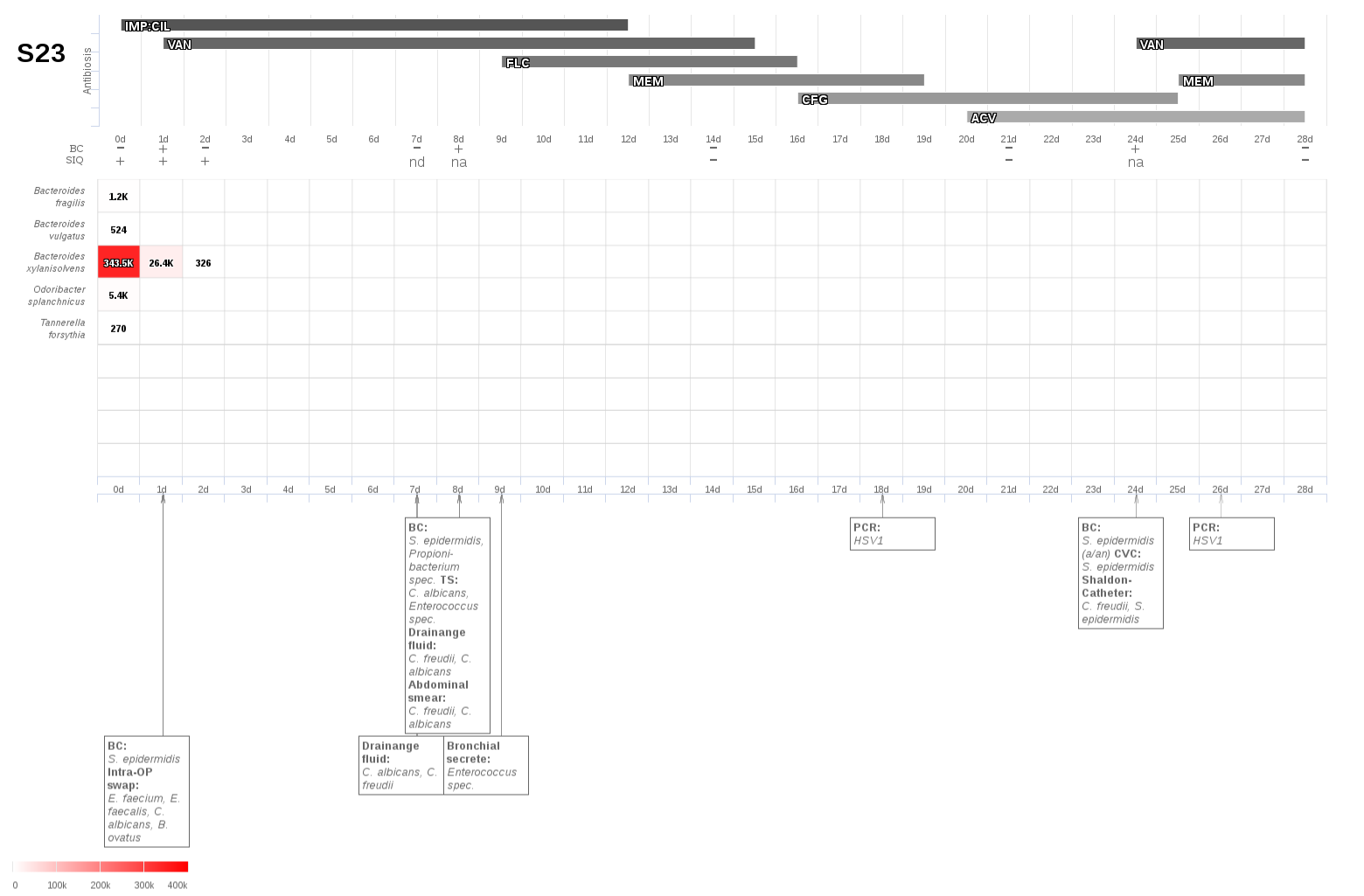
**

S24: Torsion of the intestines following a gastric bypass.

**
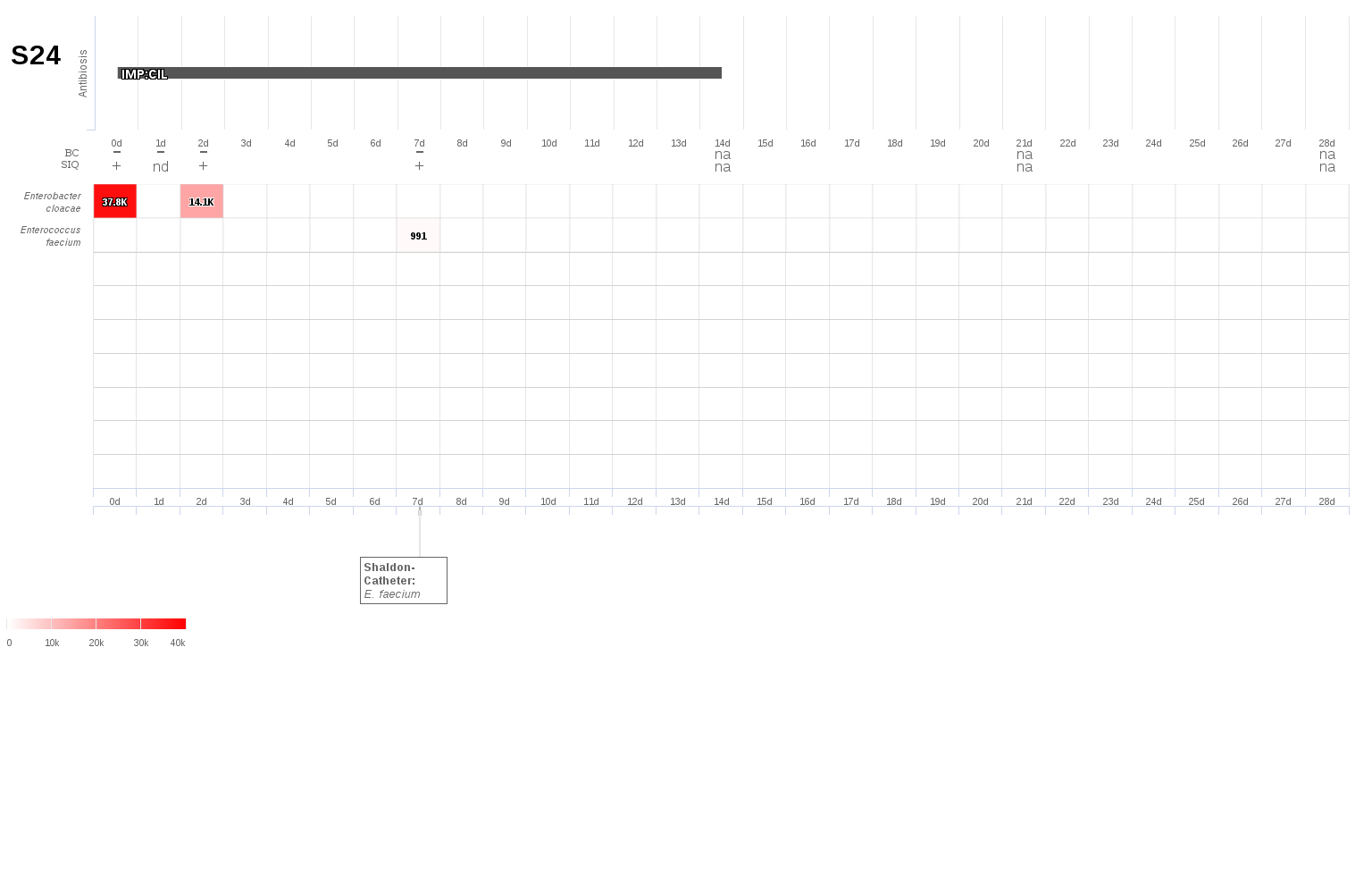
**

S25: Pleural empyema following liver resection due to a hilar cholangiocarcinoma (Klatskin tumor).

**
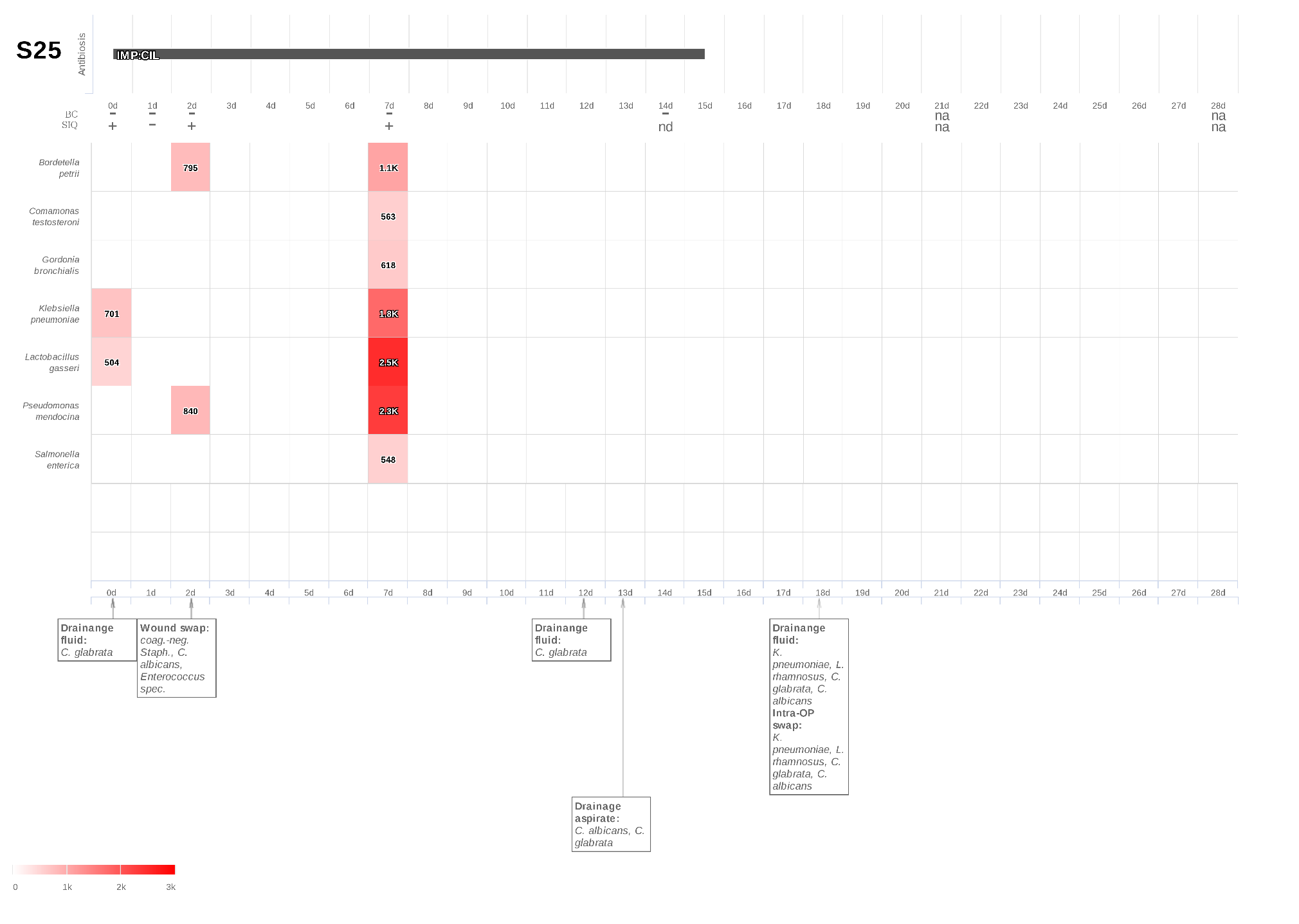
**

S26: Colon and small bowel ischemia.

**
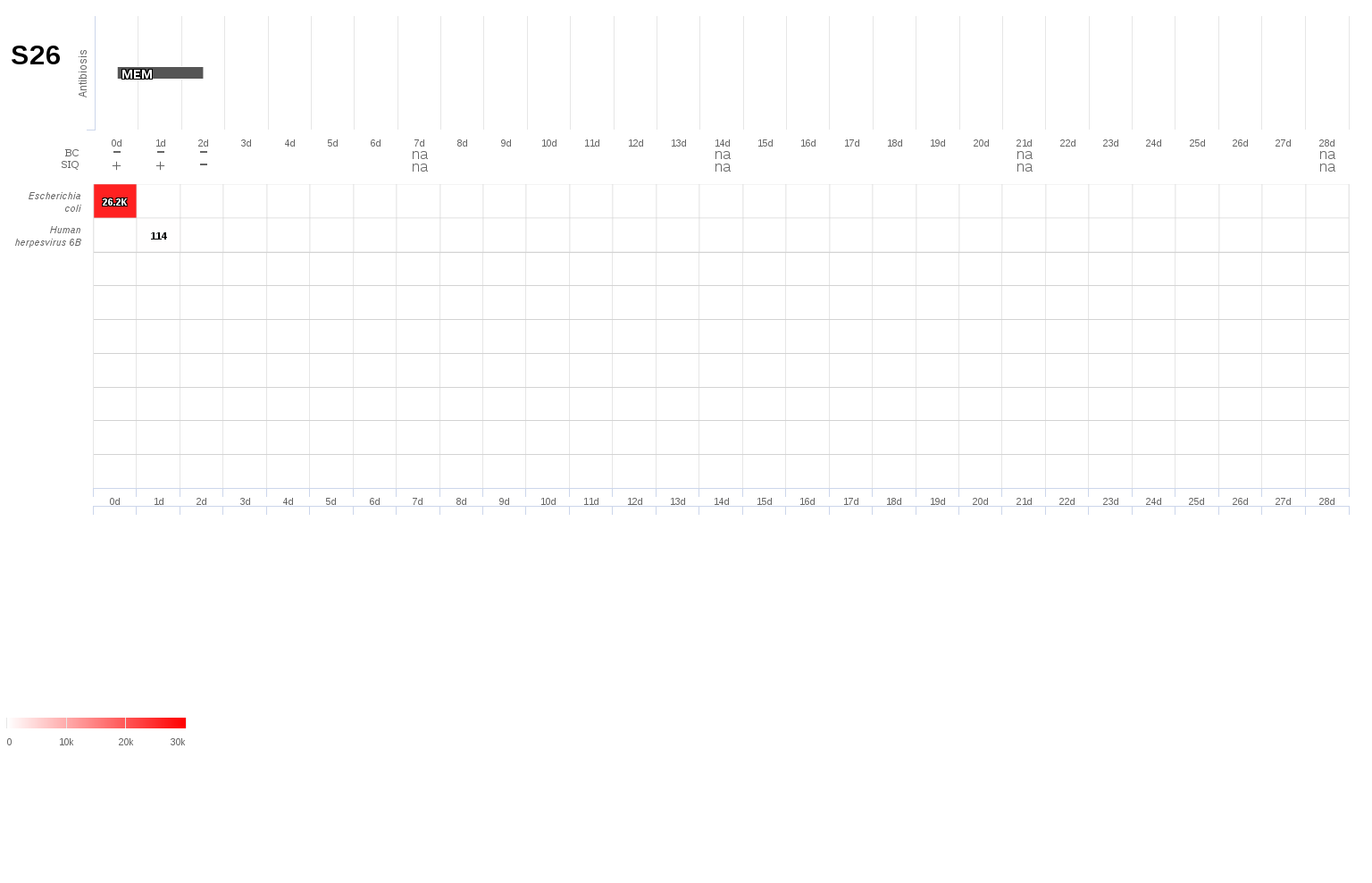
**

S27: Anastomotic insufficiency with diffuse peritonitis following proctocelectomy due to colitis ulcerosa.

**
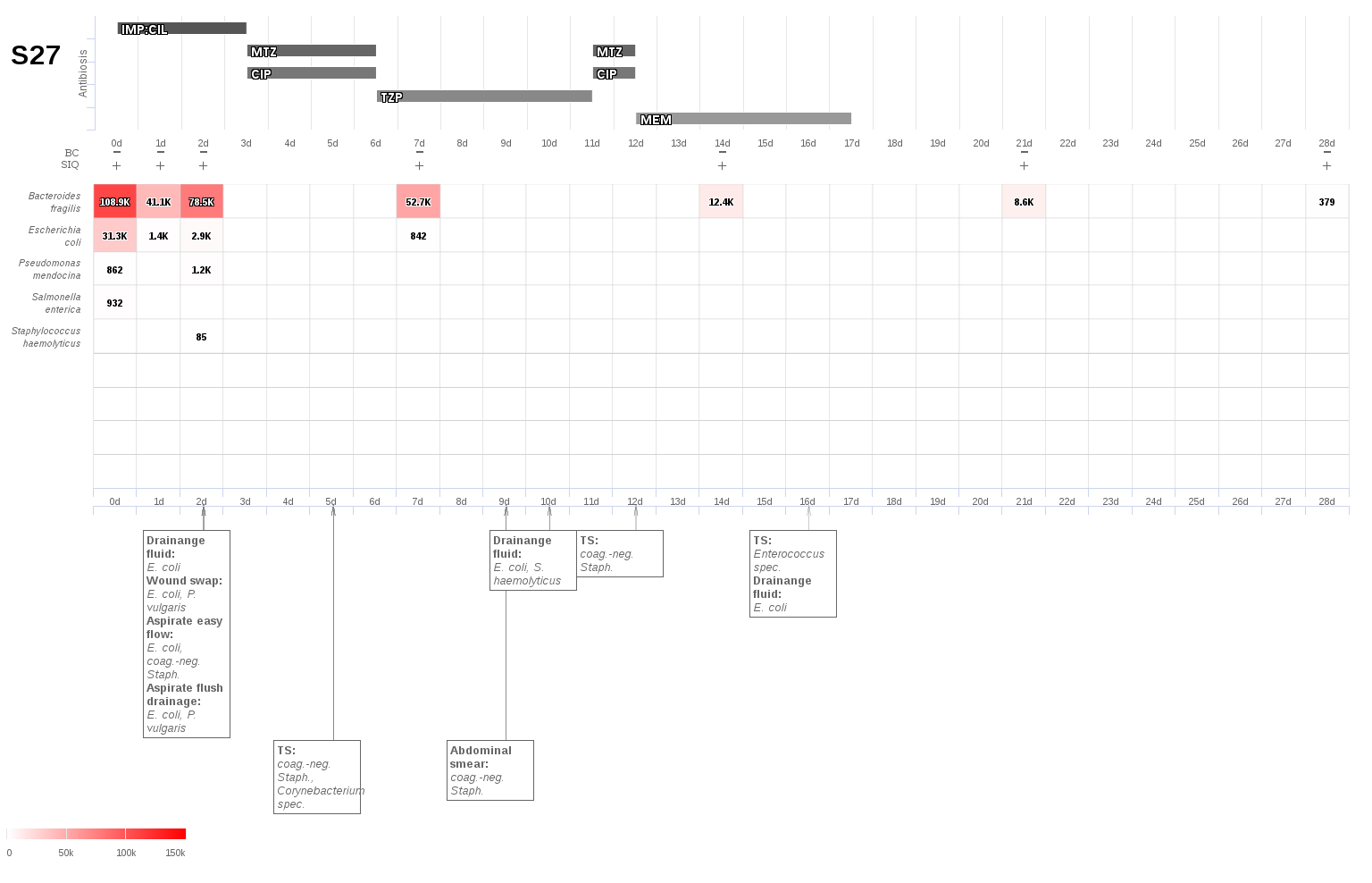
**

S29: Cecal perforation with diffuse peritonitis.

**
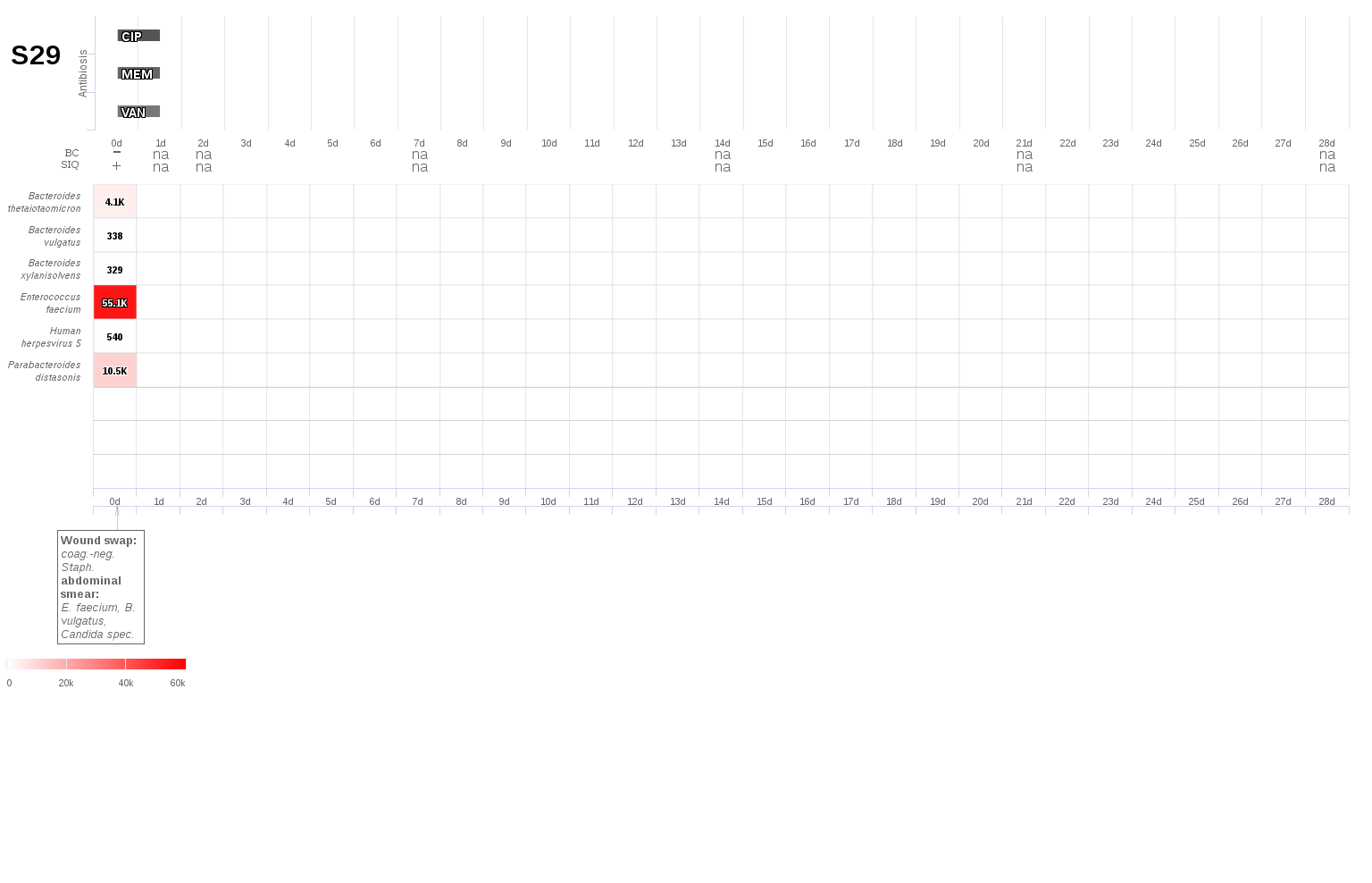
**

S30: Necrotizing pancreatitis following pancreatoduodenectomy.

**
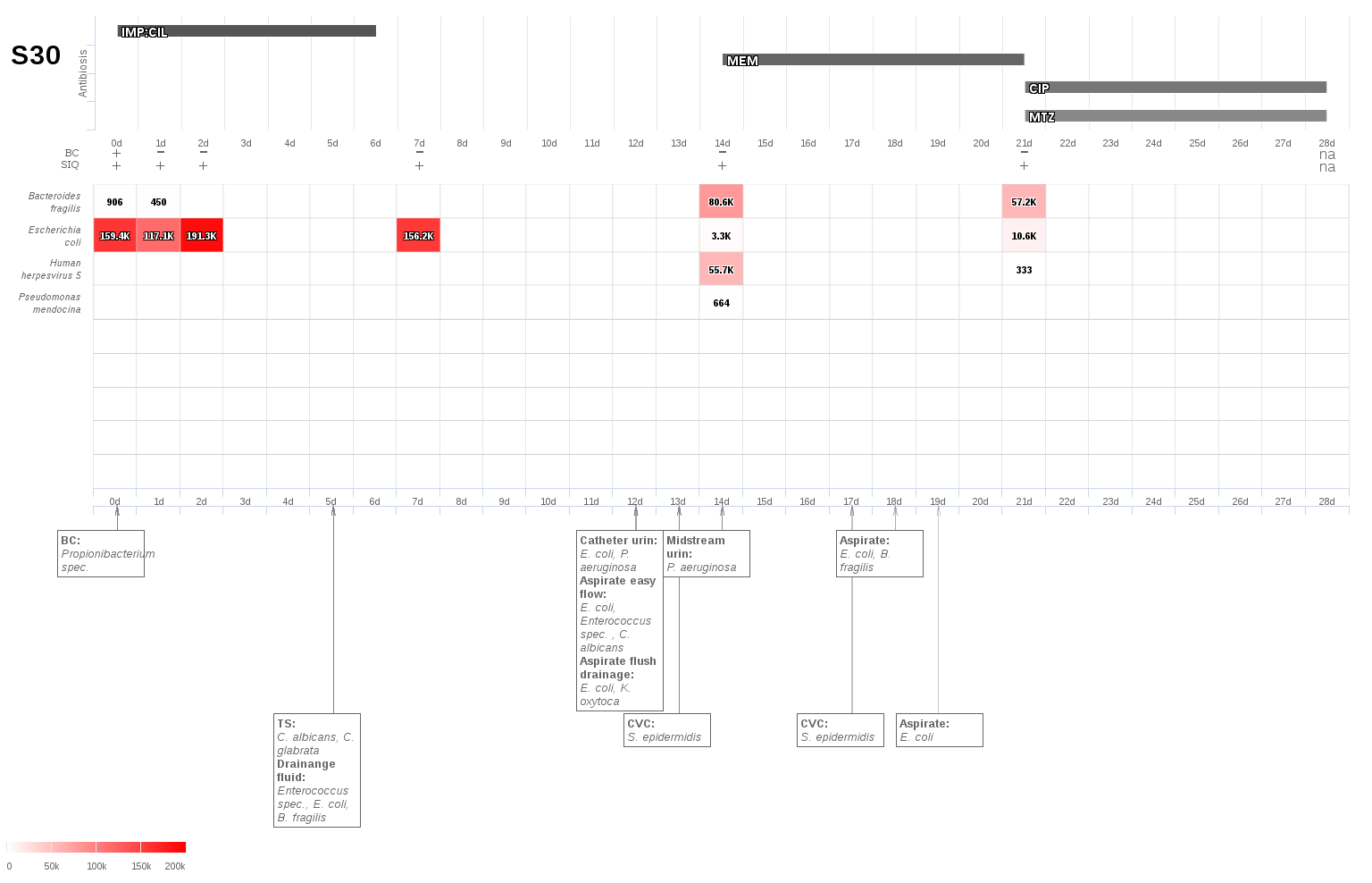
**

S32: Infected bilioma and biliary leakage following cholecystectomy due to a hepatic metastatic pancreatic cancer.

**
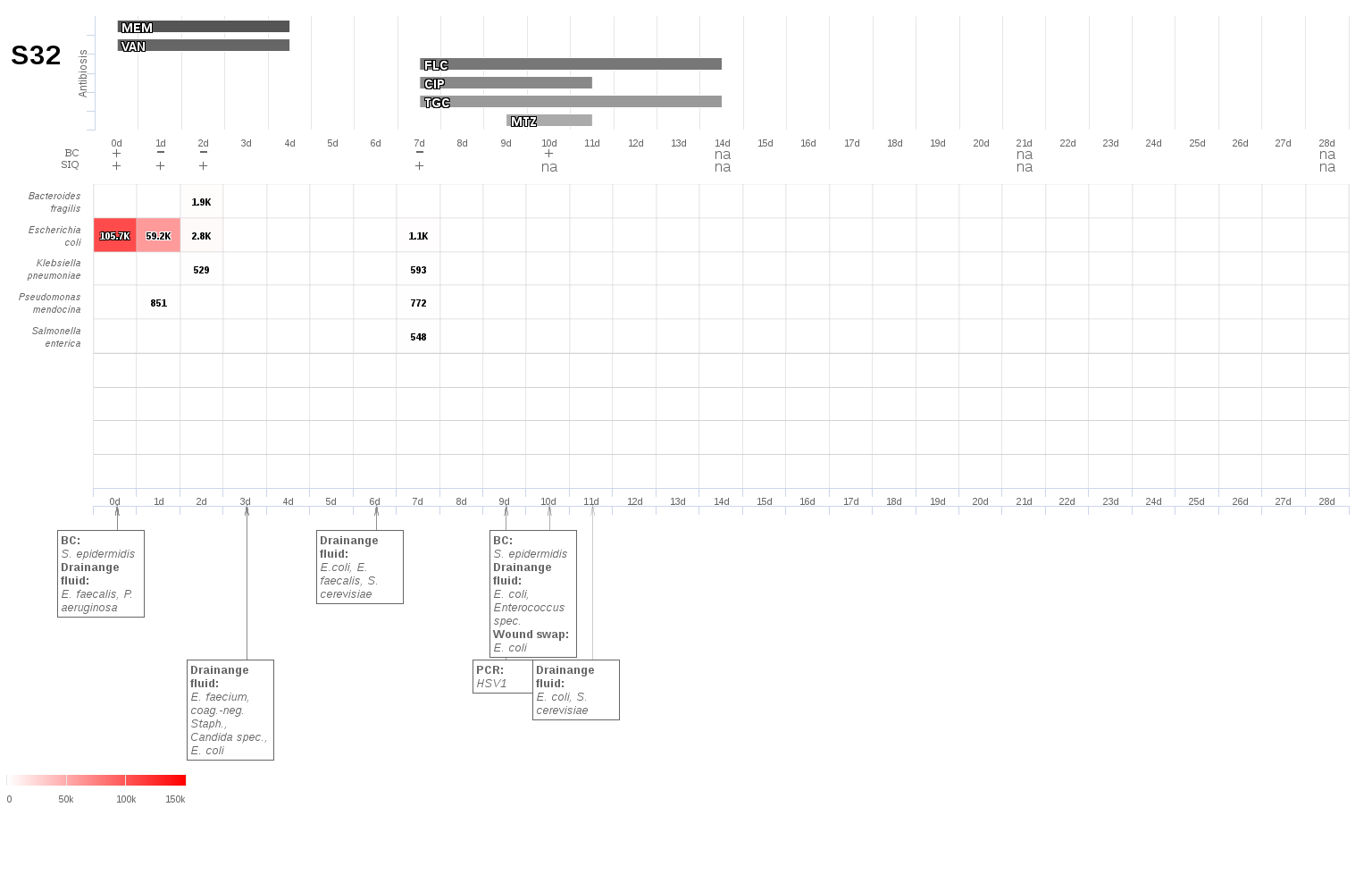
**

S33: Megacolon with perforation and diffuse peritonitis.

**
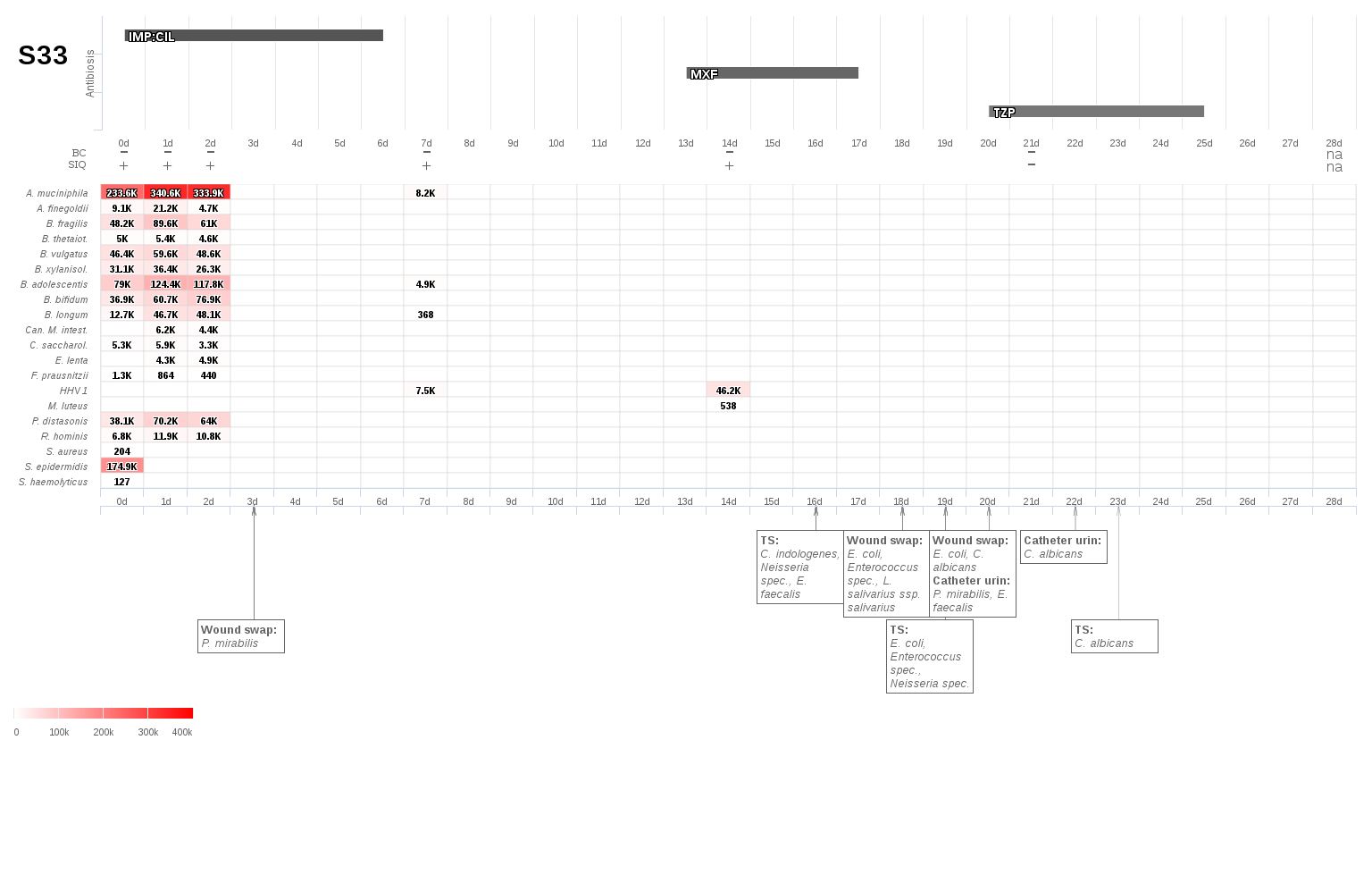
**

S34: Anastomotic insufficiency with diffuse peritonitis following hemicolectomy due to colon cancer.

**
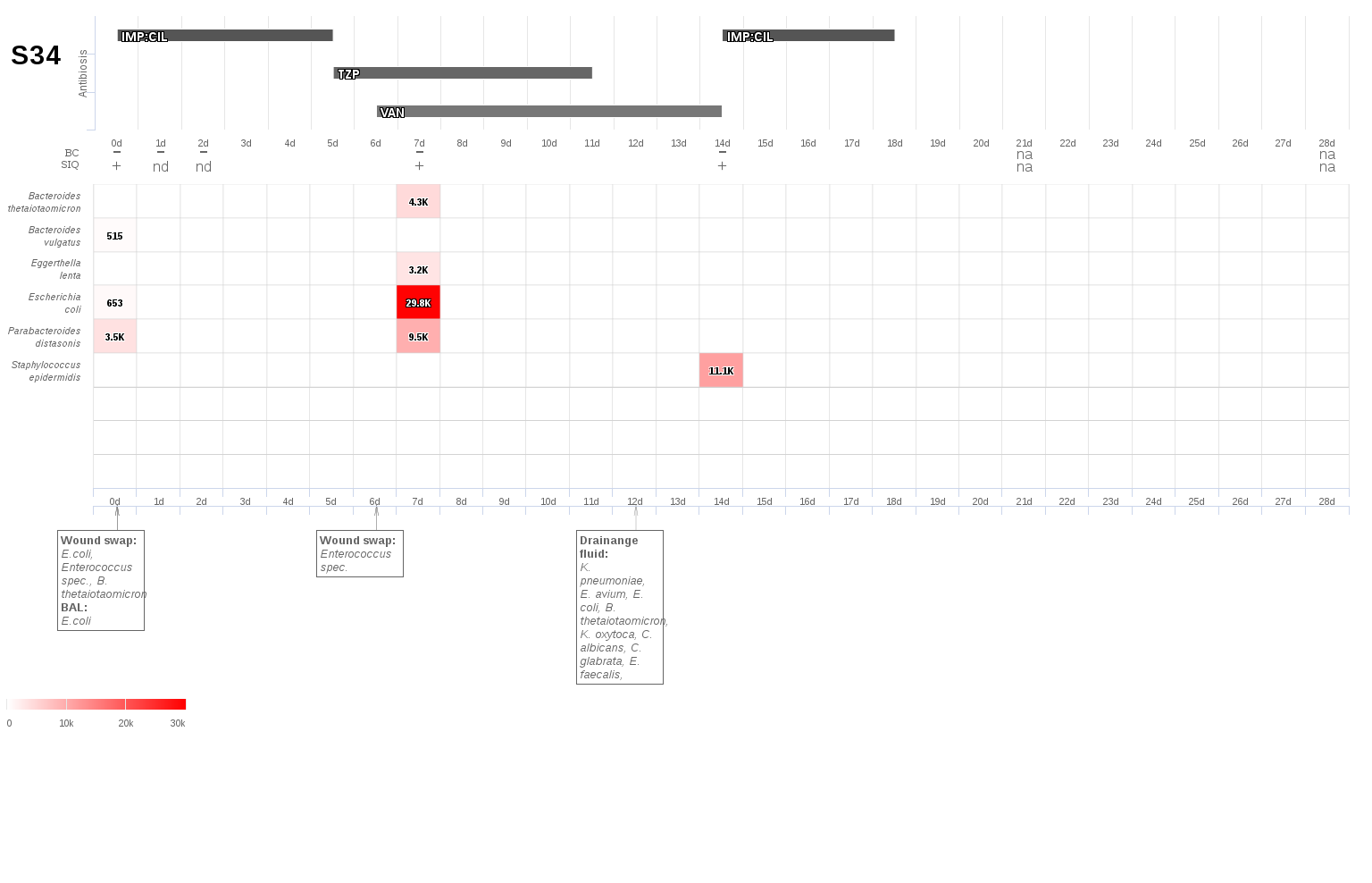
**

S35: Perforation of the colon following resection of the lower lobe of the right lung due to a liver abscess.

**
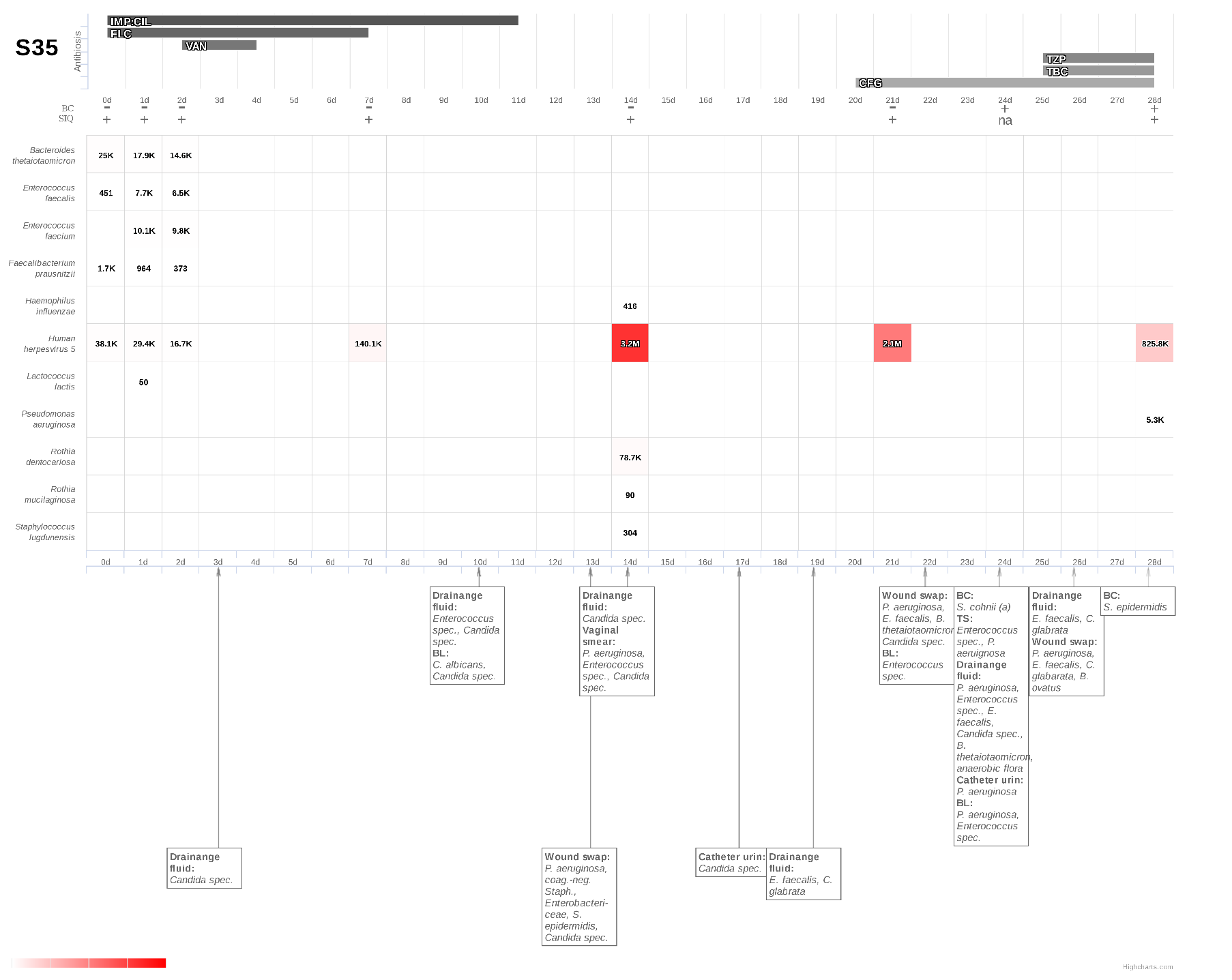
**

S37: Pneumonia and anastomotic insufficiency following distal esophagectomy due to a gastric cancer.

**
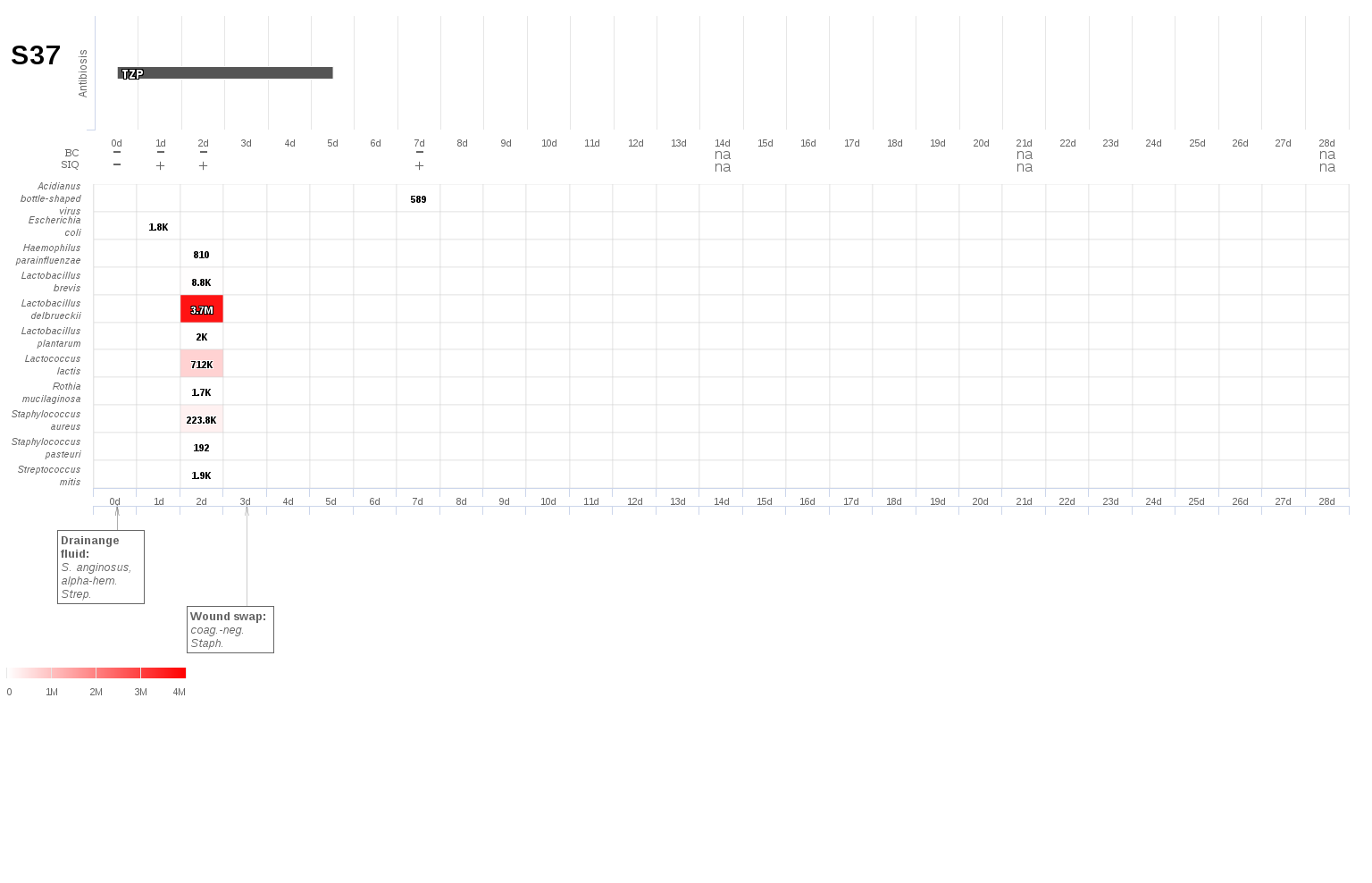
**

S38: Anastomotic insufficiency of the biliodigestive anastomosis following liver resection due to a hilar cholangiocarcinoma (klatskin tumor).

**
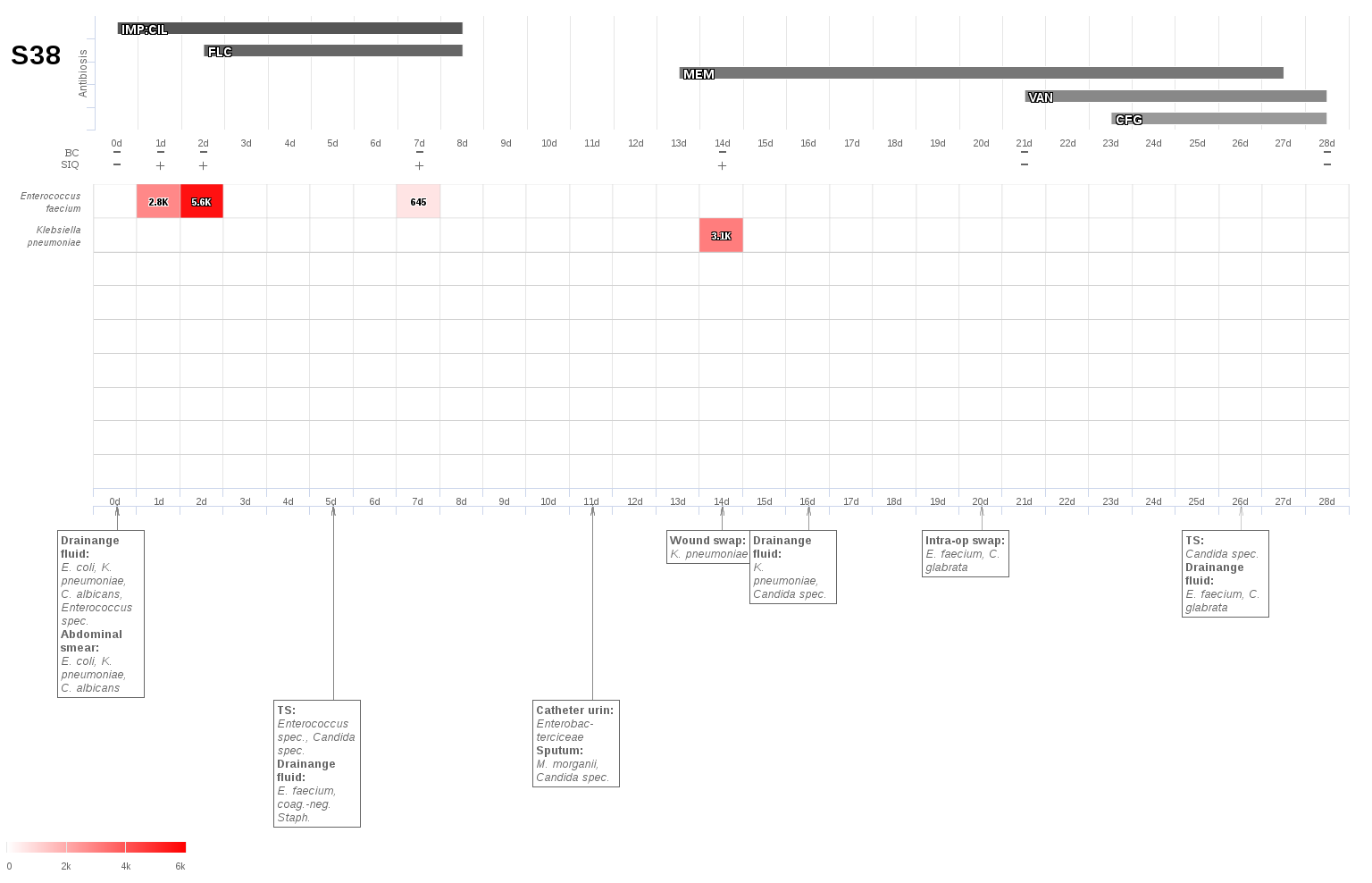
**

S39: Anastomotic insufficiency following anterior rectum resection.

**
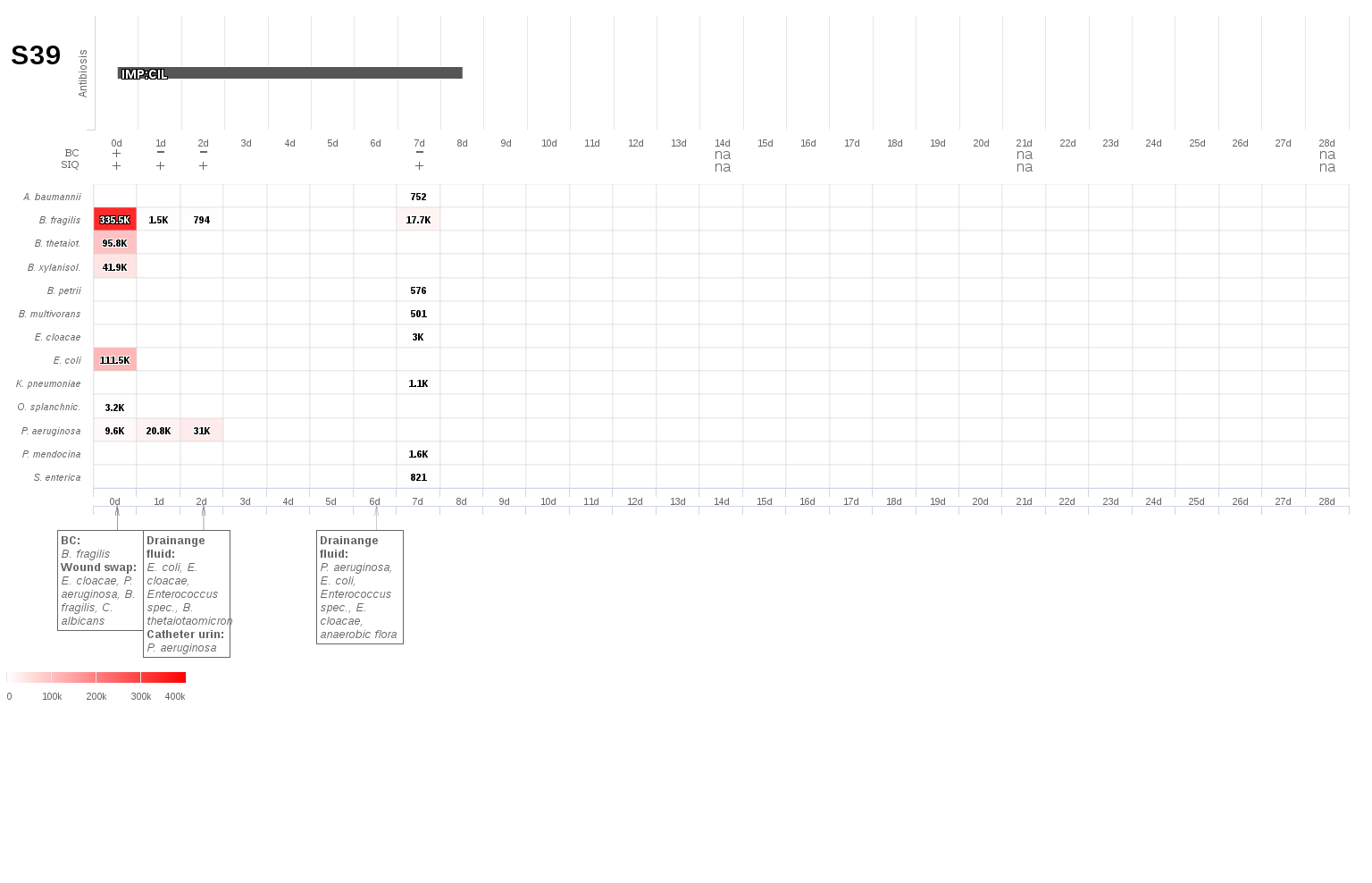
**

S41: Perforation of the colon due to multiple desmoids.

**
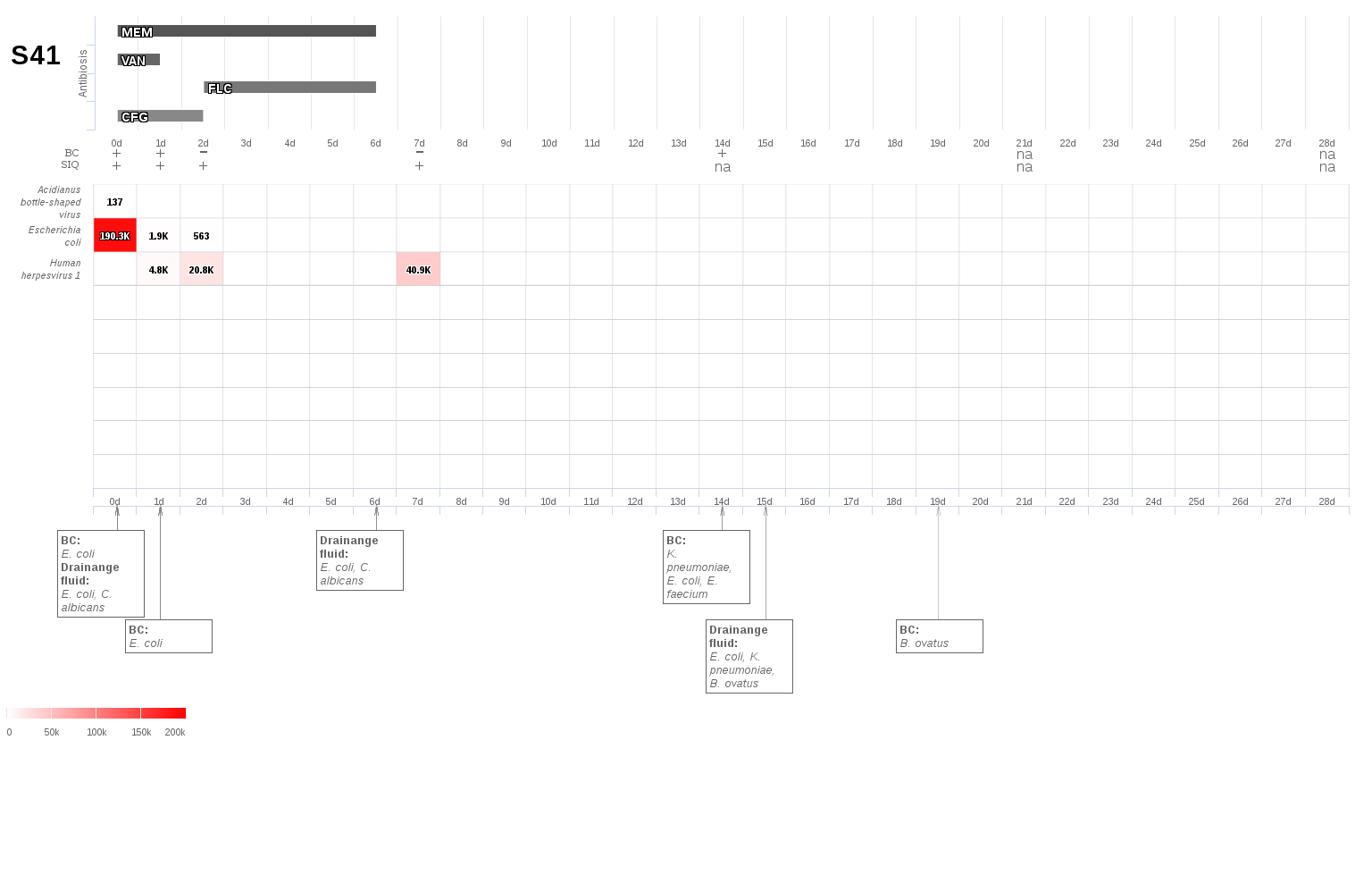
**

S42: Small bowel perforation due to a mechanical ileus with aspiration pneumonia.

**
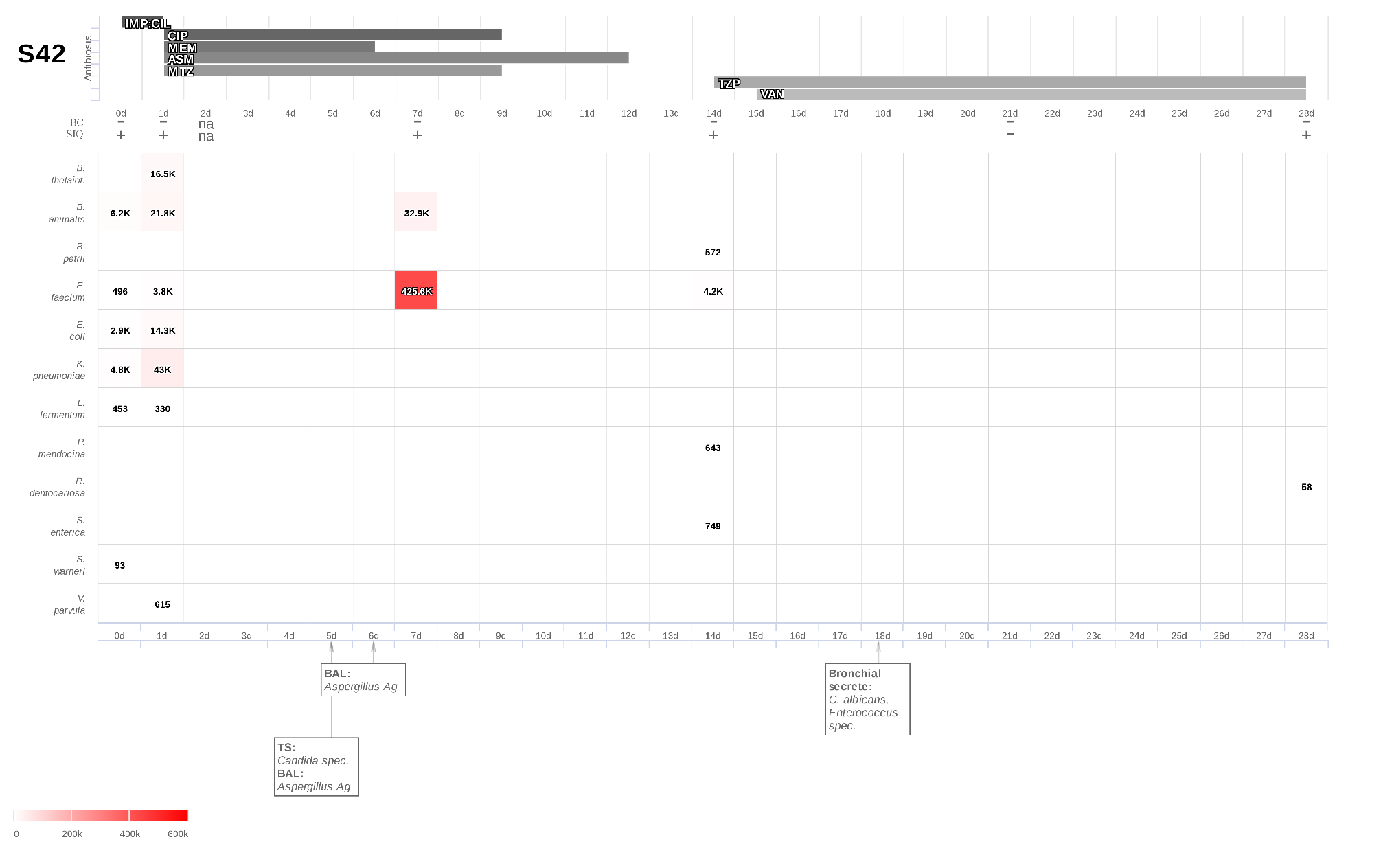
**

S43: Aspiration pneumonia due to a postoperative ileus.

**
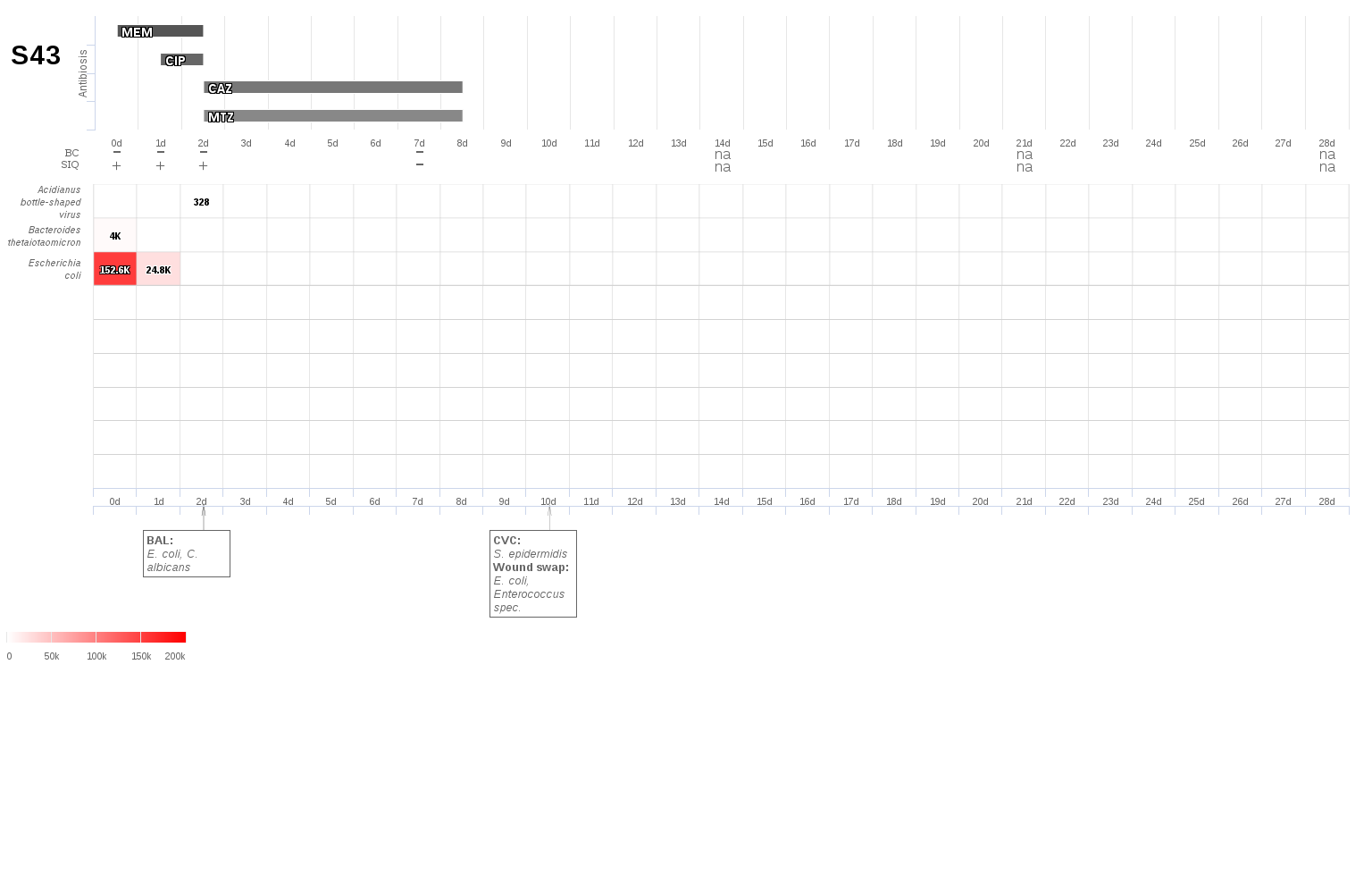
**

S44: Anastomotic insufficiency following duodenectomy due to a duodenal adenomatosis.

**
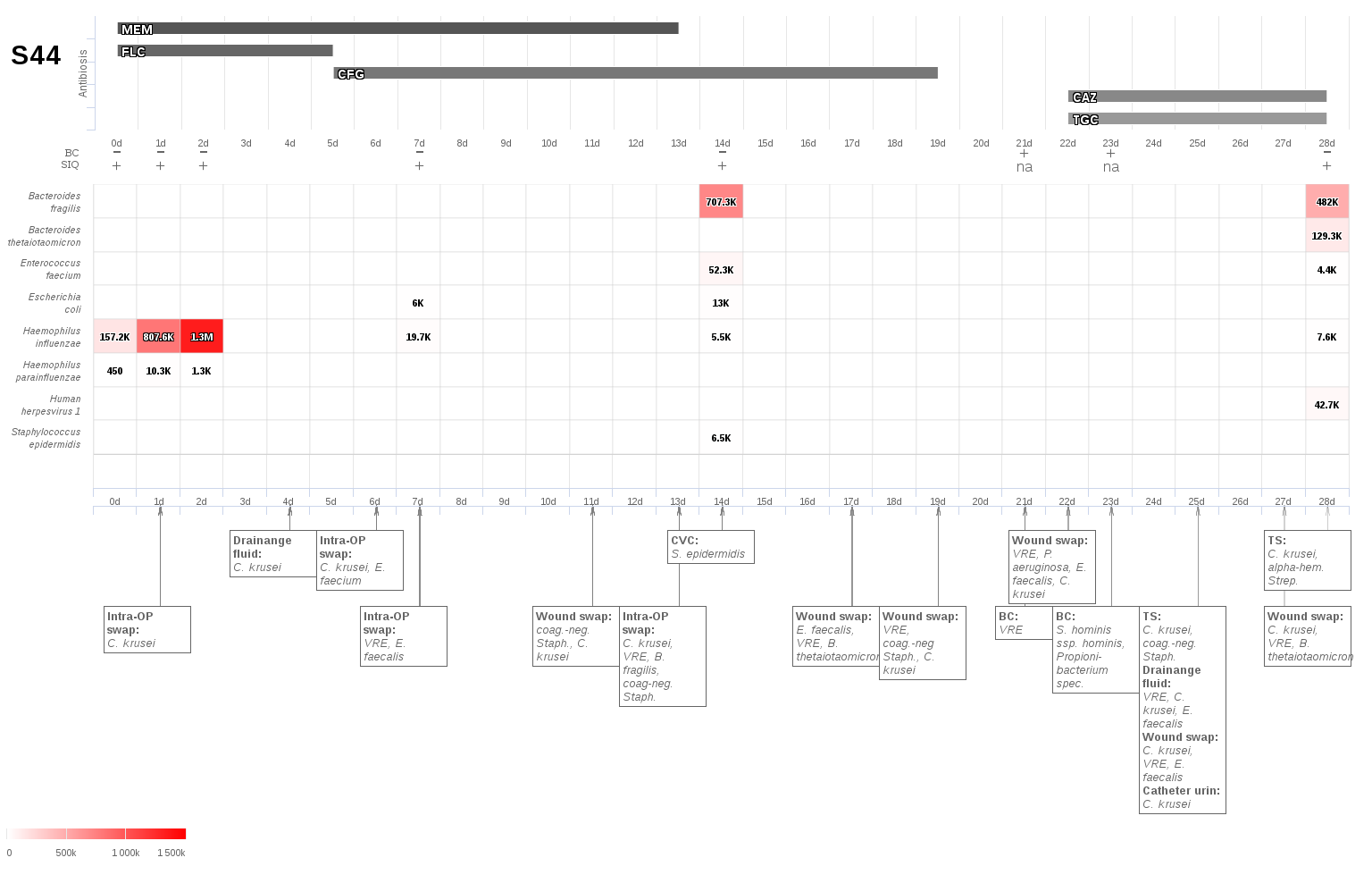
**

S45: Pneumonia following hernia repair.

**
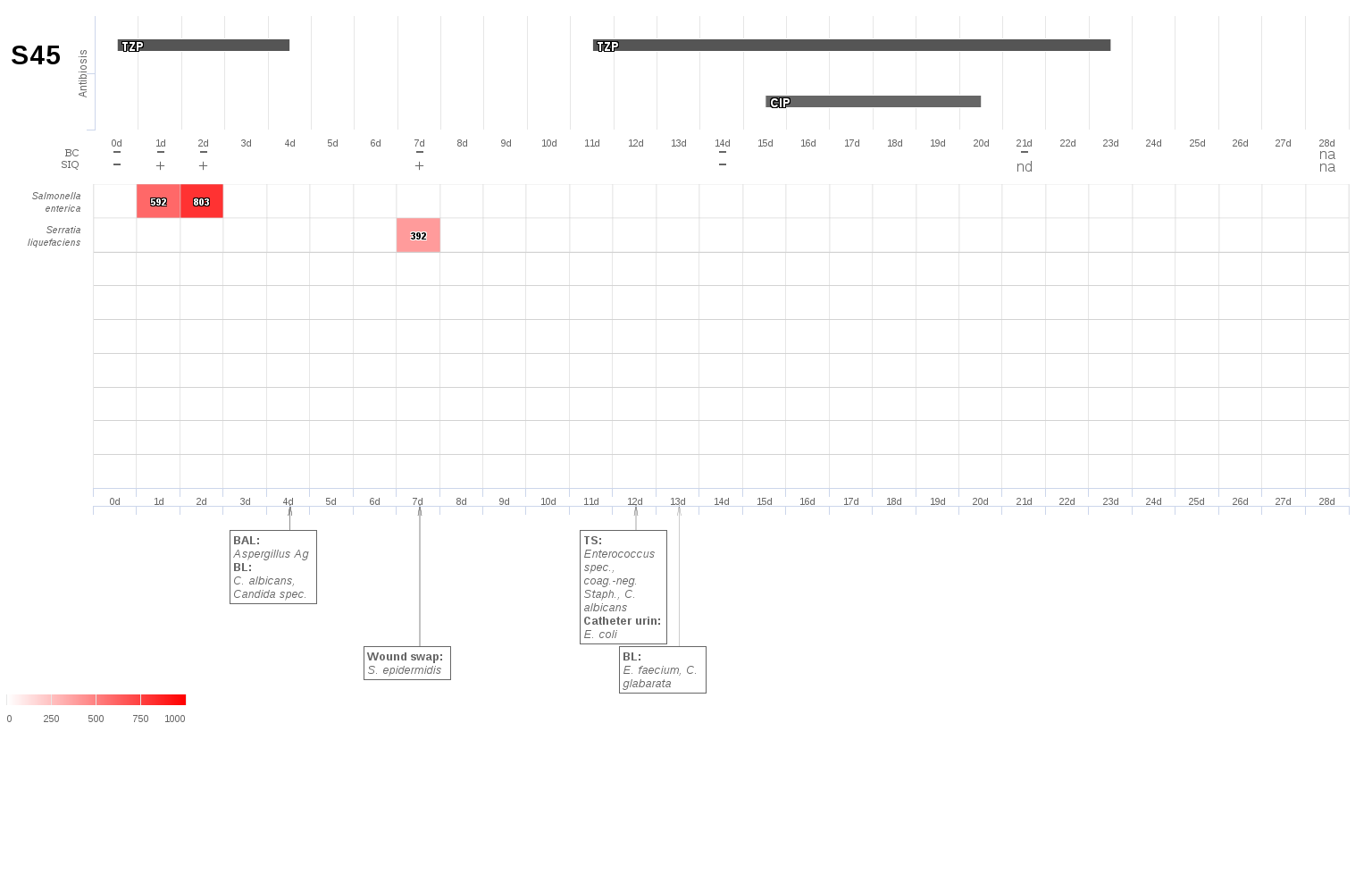
**

S46: Biliary leakage with peritonitis following thrombectomy of the inferior vena cava due to advanced renal cell cancer.

**
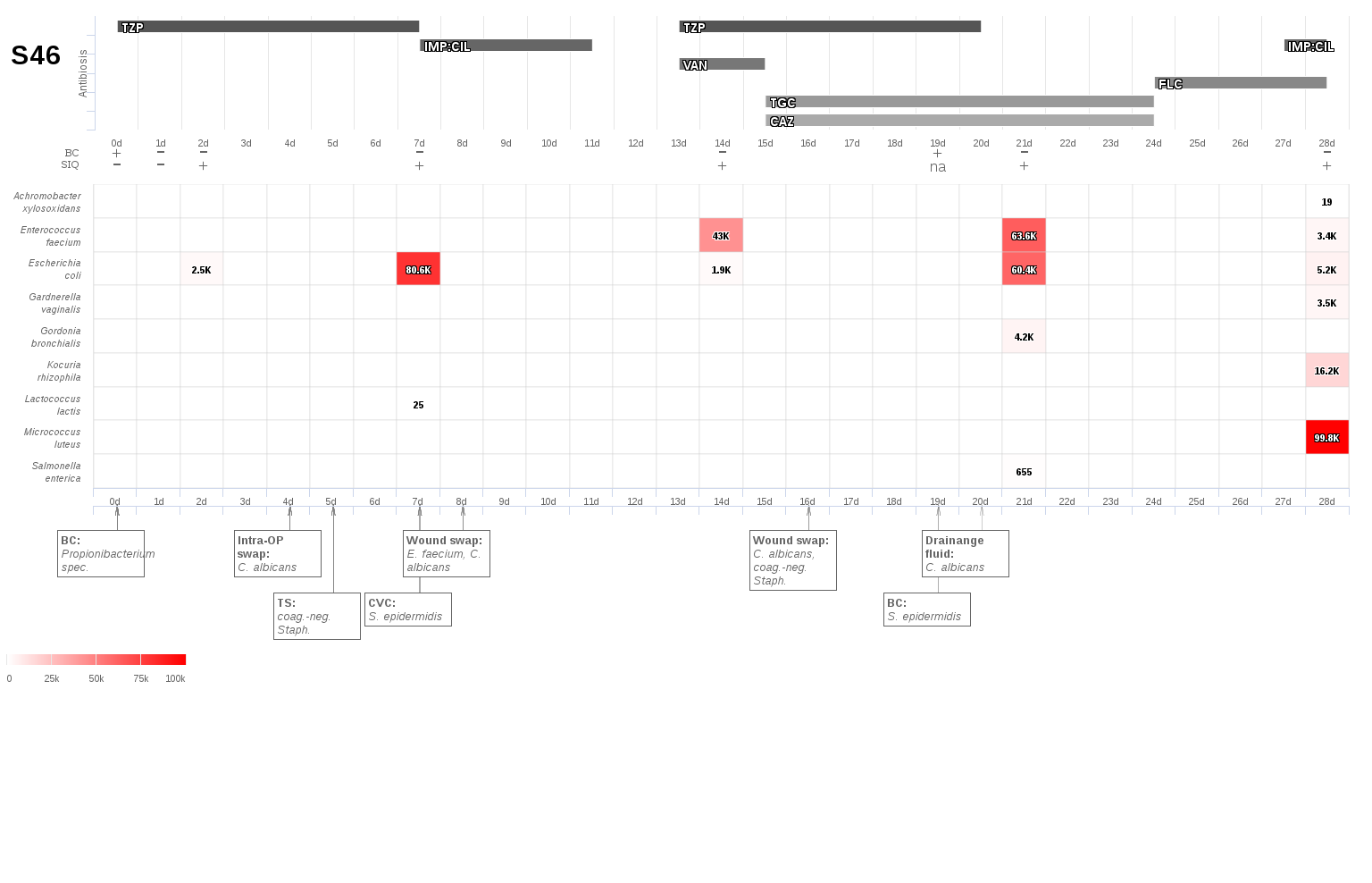
**

S47: Perforation of the colon ascendens due to colon cancer.

**
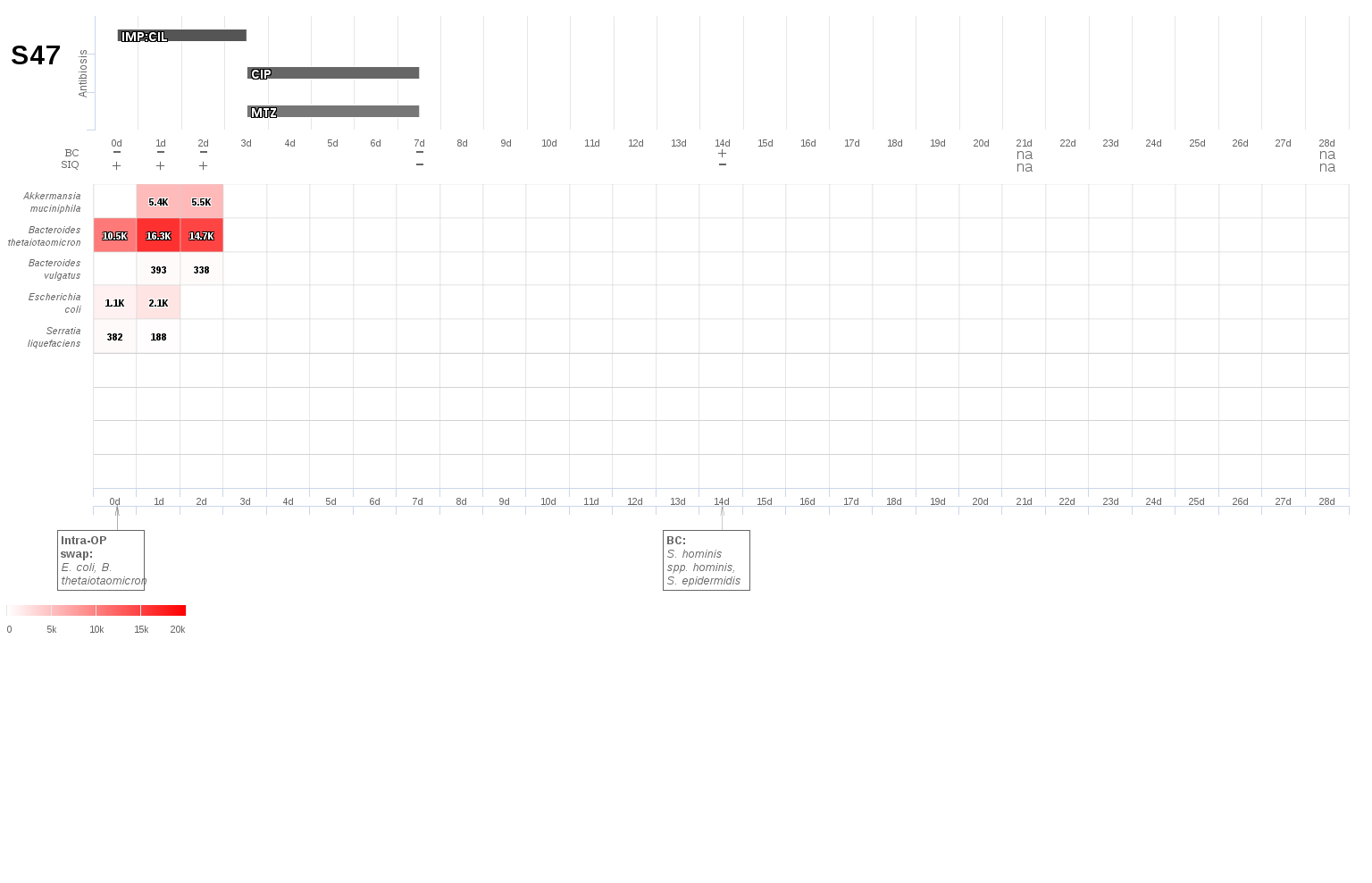
**

S48: Aspiration pneumonia following total gastrectomy.

**
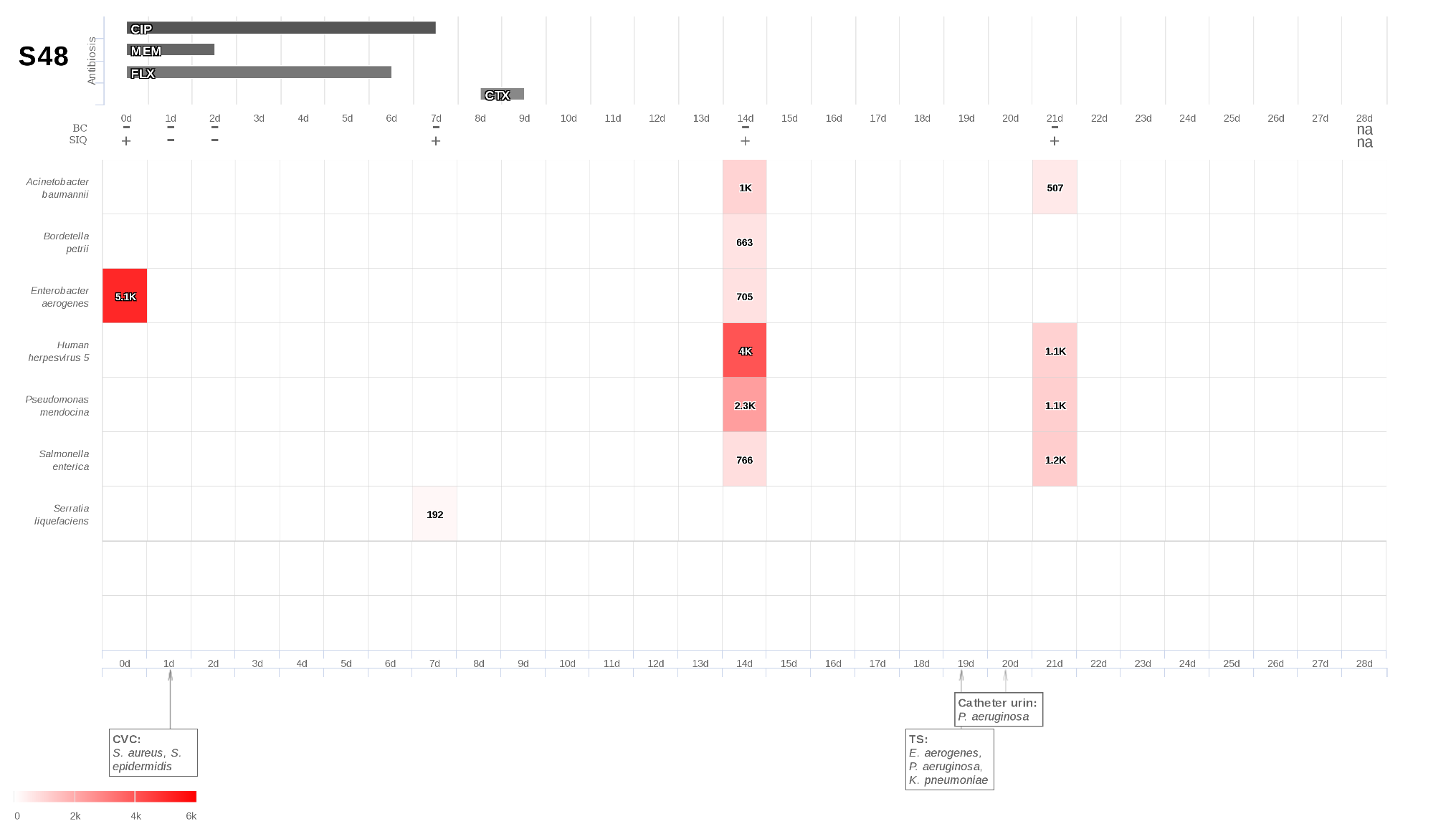
**

S49: Aspiration pneumonia due to acute necrotizing pancreatitis.

**
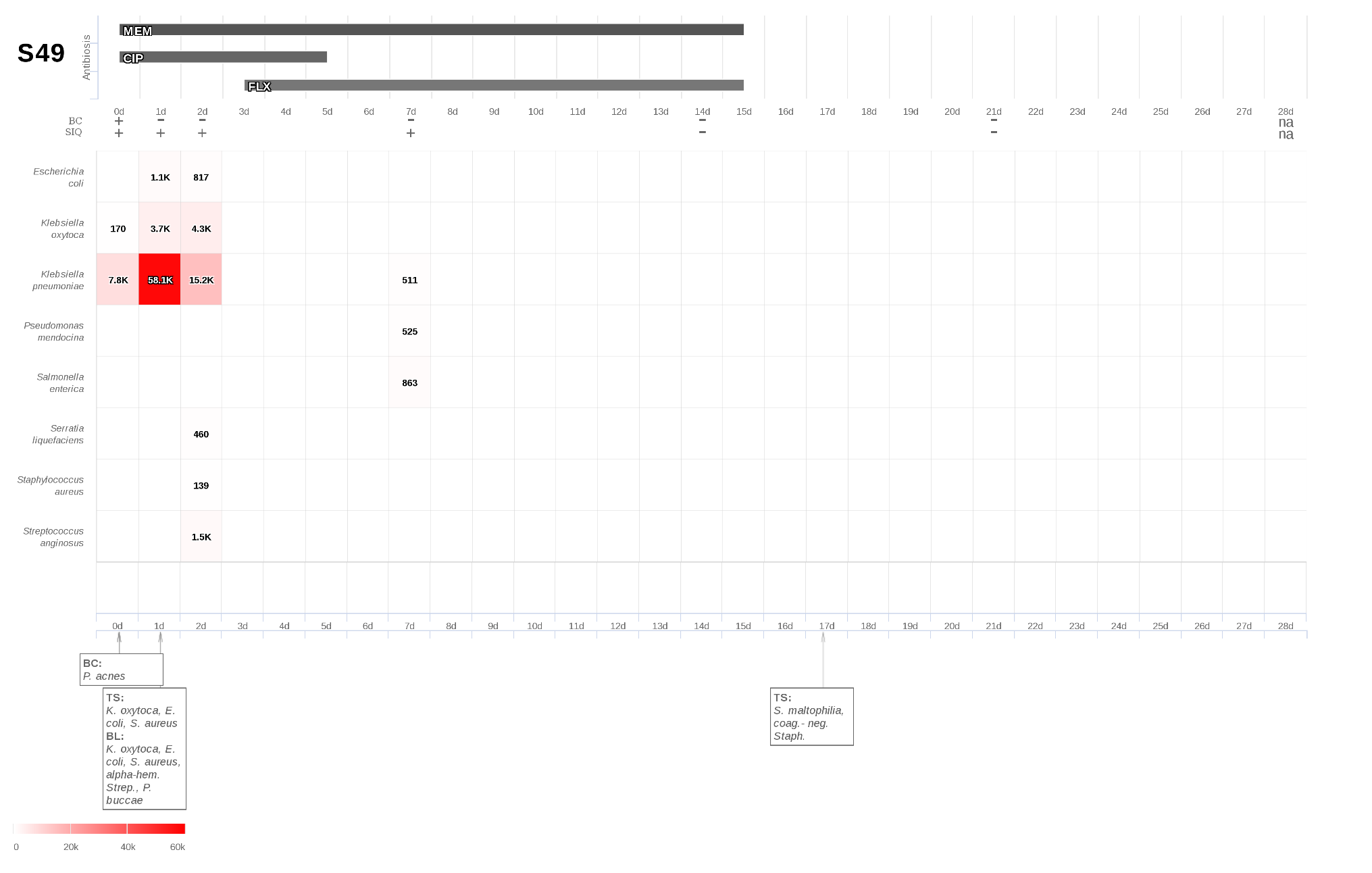
**

S50: Anastomotic insufficiency following anterior rectum resection.

**
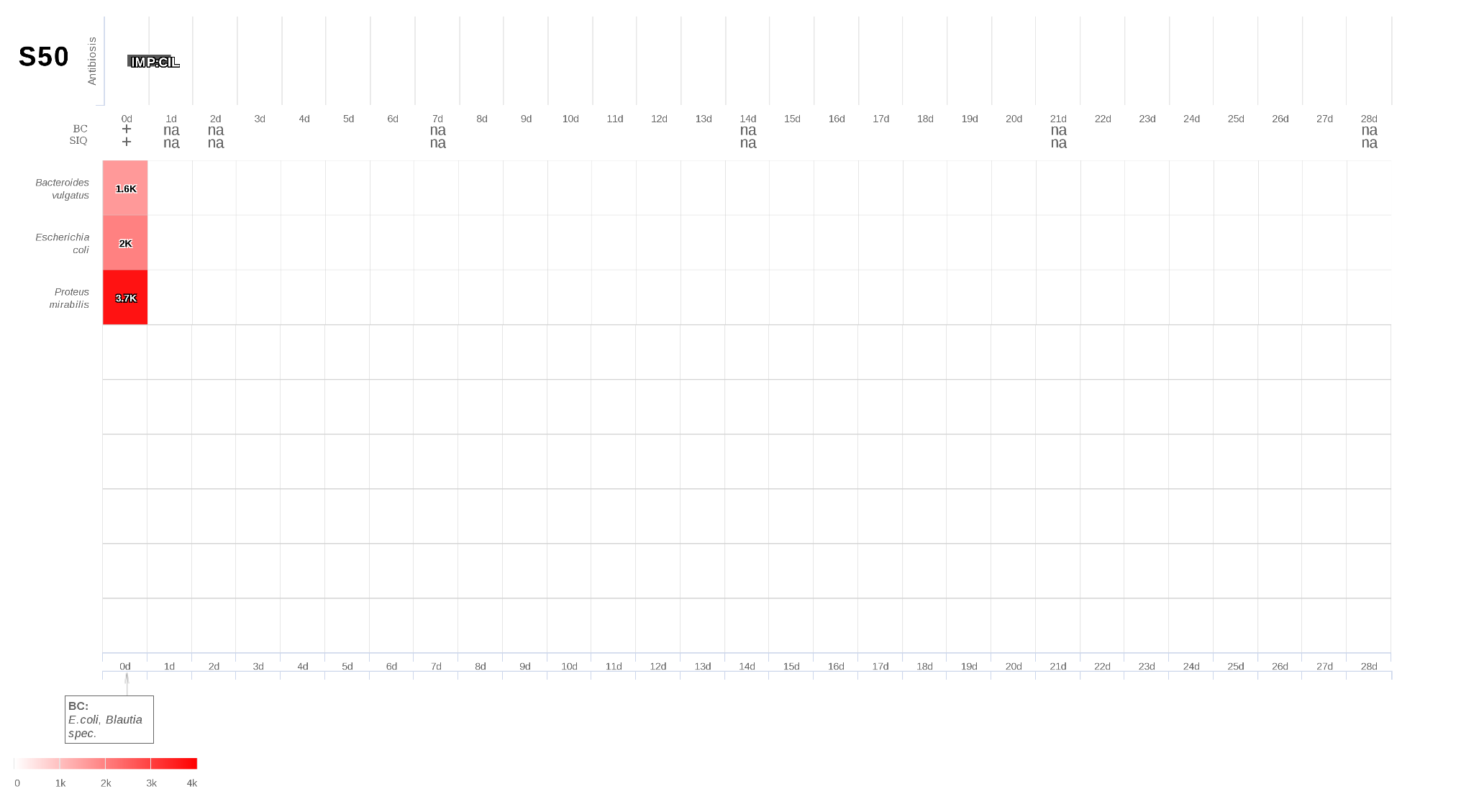
**

S51: Perforation of the small bowels following hysterectomy.

**
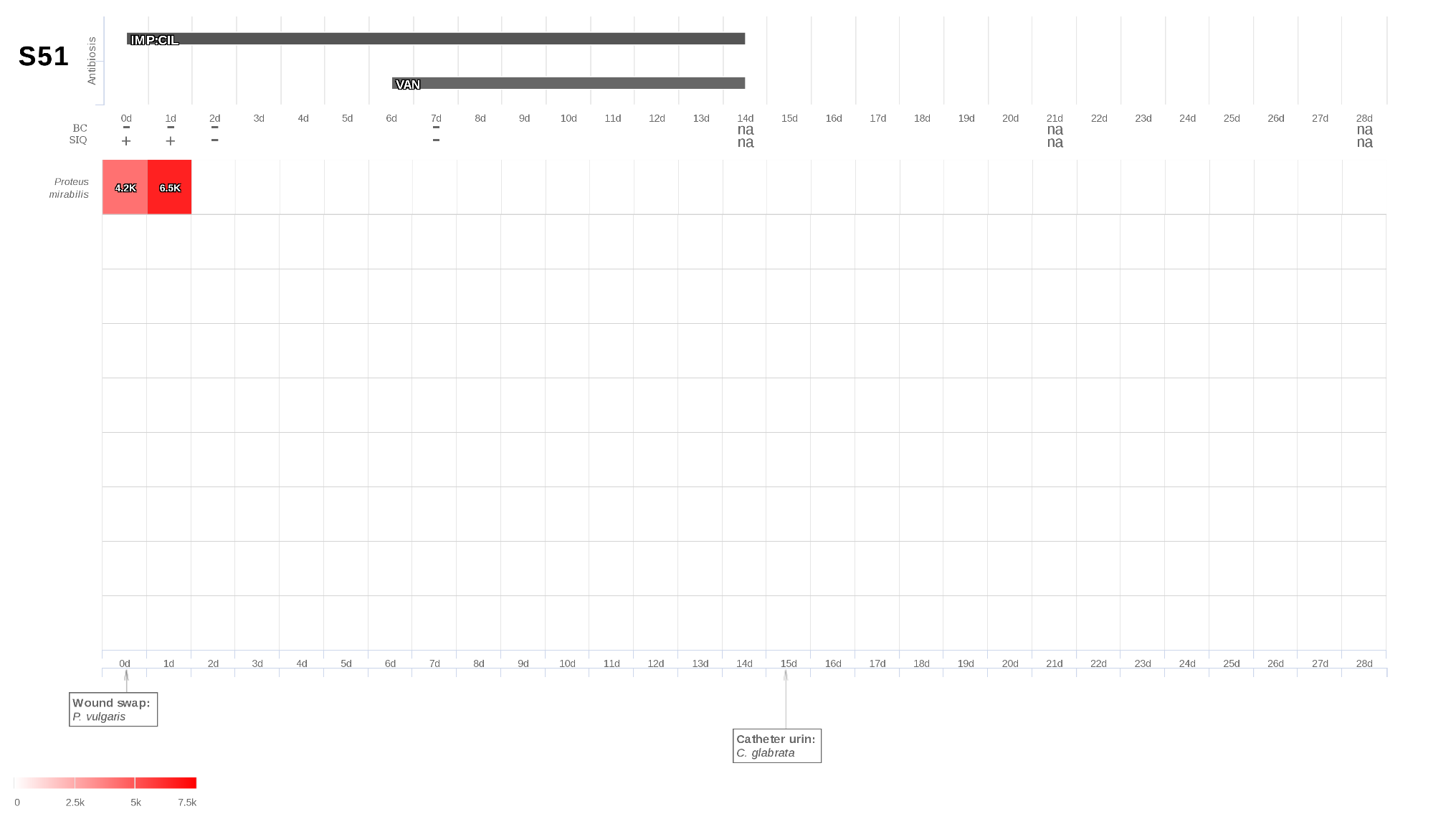
**

S52: Aspiration pneumonia due to a mechanical ileus

**
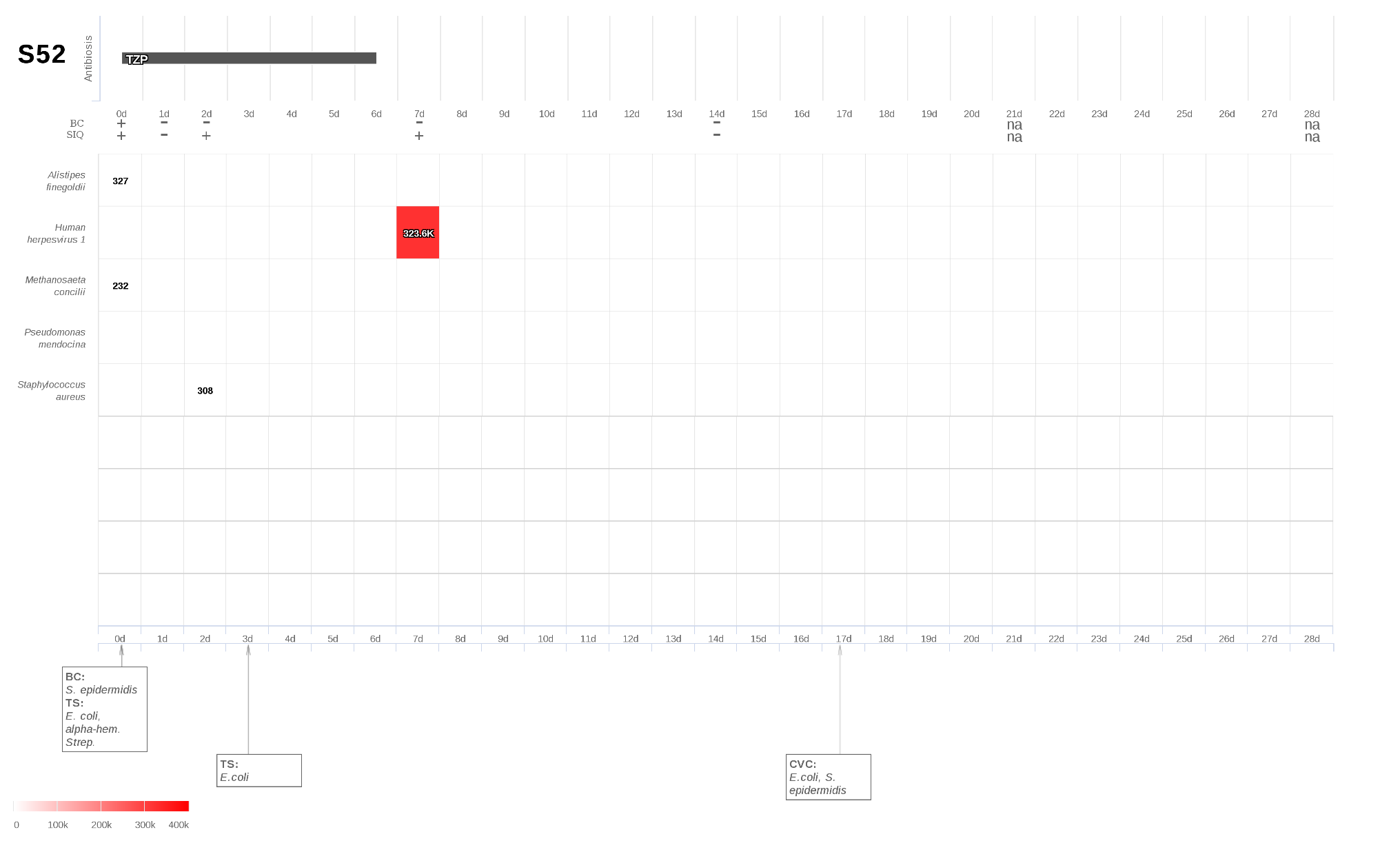
**

S53: Retroperitoneal abscess and necrotizing fasciitis due to sigmoid perforation.

**
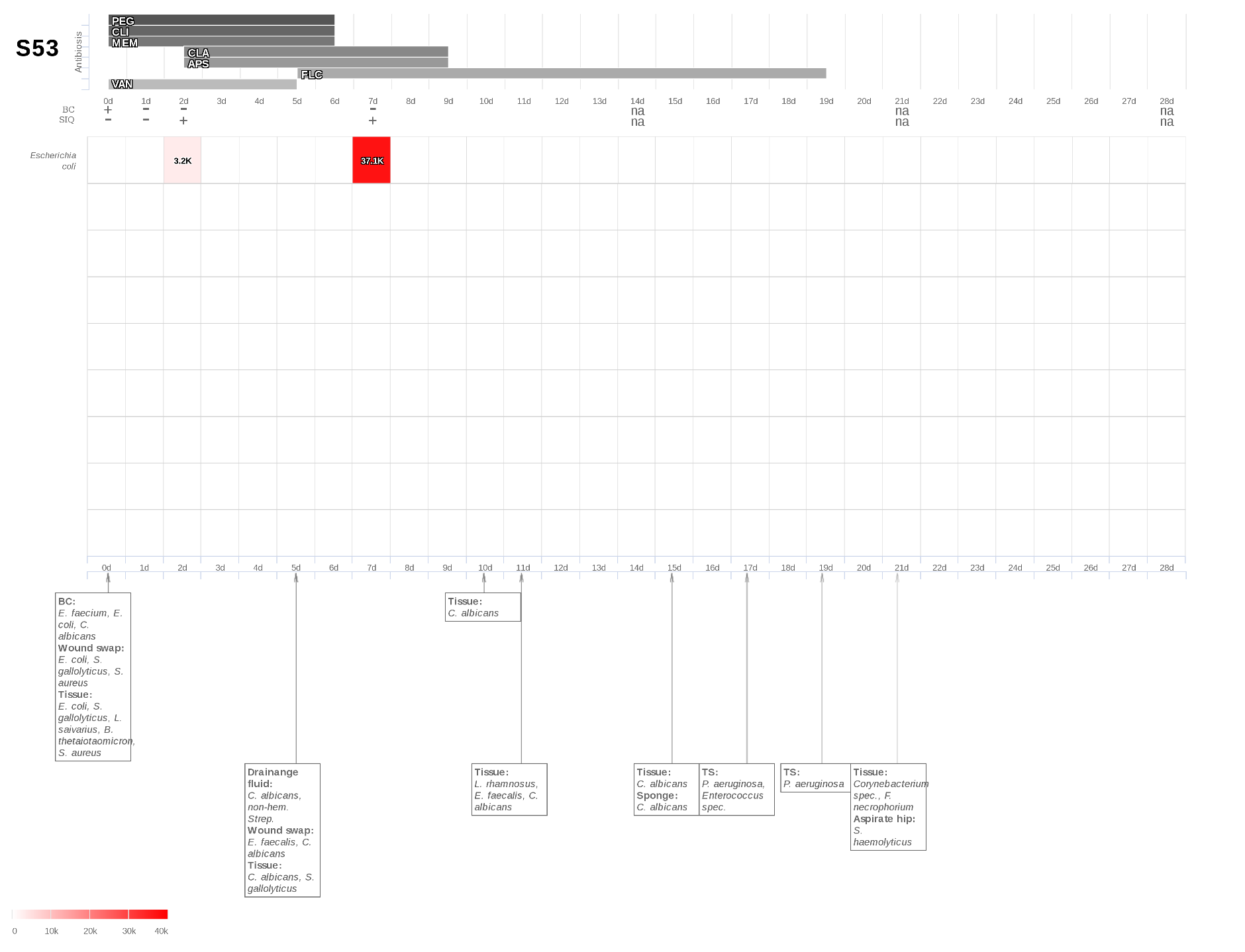
**
